# Proteomic-based identification of novel EV-derived protein antibodies biomarkers for melioidosis diagnosis

**DOI:** 10.1371/journal.pntd.0013543

**Published:** 2025-09-24

**Authors:** Nini Luo, Jun Tan, Xuemiao Li, Yanshuang Wang, Ting Zhang, Chen Chen, Lin Liu, Xinyi Song, Hua Pei, Bo Wang, Qi Li, Shen Tian, Nan Zhang, Wei Cheng, Qianfeng Xia

**Affiliations:** 1 NHC Key Laboratory of Tropical Disease Control, School of Life Sciences and Medical Technology, Hainan Medical University, Haikou, Hainan, China; 2 The Second Affiliated Hospital of Hainan Medical University, Haikou, Hainan, China; 3 Central Laboratory, Hainan General Hospital, Hainan Affiliated Hospital of Hainan Medical University, Haikou, Hainan, PR China; 4 The First Affiliated Hospital, Hainan Medical University, Haikou, Hainan, China; 5 The Center for Clinical Molecular Medical Detection, The First Affiliated Hospital of Chongqing Medical University, Chongqing, P.R. China; Yale University School of Medicine, UNITED STATES OF AMERICA

## Abstract

Melioidosis, caused by *Burkholderia pseudomallei* (*Bp*), is a life-threatening disease characterized by diverse clinical manifestations and limited diagnostic capabilities. Extracellular vesicles (EVs) have emerged as critical carriers of novel antibody targets for serodiagnosis. In this study, we established a *Bp*-infected BEAS-2B cell model (*Bp*/BEAS-2B) and isolated EV from both *Bp* and *Bp*/BEAS-2B cells to generate EV proteome, identifying potential antigenic biomarkers for melioidosis diagnosis. Bioinformatics analysis identified PPEP and POMCR proteins as candidate antigens, with BLF1 and omp A serving as positive controls. Using a self-developed IgM-ELISA, serum samples from 43 melioidosis patients and 47 healthy volunteers were analyzed to detect antibodies against these antigens. Anti-POMCR IgM demonstrated exceptional diagnostic performance, with an AUC of 0.9872 (95% CI: 0.9713-1.003), sensitivity of 93.02% and specificity of 97.92% at a cutoff value of OD_450_ = 0.118. Similarly, IgM against PPEP, BLF1, and omp A also showed high diagnostic accuracy, with AUC values of 0.969, 0.9621, and 0.976, respectively. The accuracy of anti-POMCR and anti-PPEP were 96.43% and 95.54%, respectively, equivalent to anti-omp A (93.75%) and anti-BLF1 (91.96%). Antibodies to EV-derived proteins effectively differentiated melioidosis patients from other bacterial infections and healthy volunteers, highlighting their clinical potential as diagnostic tools for melioidosis.

## Author summary

Melioidosis, caused by *Burkholderia pseudomallei* (*Bp*), is a life-threatening disease with limited diagnostic options. In this study, we discovered that IgM antibodies against EV-derived proteins serve as highly accurate serological biomarkers. Using EVs isolated from *Bp*-infected BEAS-2B cells, we identified two antigens PPEP and POMCR. In 90 clinical sera (43 patients/47 controls), anti-POMCR IgM achieved 93.0% sensitivity and 97.9% specificity via ELISA – surpassing current methods. Crucially, these EV-targeting antibodies differentiated melioidosis from other bacterial infections, highlighting their clinical potential as diagnostic tools for melioidosis.

## Introduction

Melioidosis is a highly fatal tropical infectious disease caused by *Burkholderia pseudomallei* (*Bp*), widely distributed in tropical regions with diverse clinical manifestations. In China, it is clustered in Hainan, Leizhou, and Guangxi (>50/100,000 incidence), with emerging foci in Fujian and Taiwan. Outbreaks peak post-typhoon (Jun–Oct) under >28°C/80% humidity, tripling the risk of infection. Farmers and diabetics (30–70Y) are predominantly affected, with infections occurring via skin (68%) or aerosols (24%). In endemic regions, the mortality rate ranges from 19% to 36%, with acute septicemia cases accounting for approximately 60%, and the case fatality rate exceeding 30%. Because *B. pseudomallei* can persist intracellularly after antibiotic treatment, recurrence of acute infection occurs in ~2% of cases. True chronic infections (symptomatic persistence >2 months) are rare but may persist for years to decades. [1]. Its classification as a WHO Category B bioweapon and CDC Tier 1 pathogen [2]. Underscores the critical need for early diagnosis – yet significant limitations persist across current diagnostic modalities. However, traditional culture methods suffer from insufficient sensitivity and specificity, limiting their diagnostic value. Molecular techniques like PCR and RNA sequencing [3–6], while identifying nucleic acid biomarkers (e.g., IL-1R2, GAS7), require stringent bacterial DNA extraction and primer design, hampering field deployment. In contrast, protein biomarkers offer prognostic value due to their direct involvement in disease progression and can be used to diagnose melioidosis or assess its severity [7]. Therefore, identifying more specific protein biomarkers in patient serum is crucial for diagnosing melioidosis.

In recent years, numerous studies have shown that extracellular vesicles (EVs) offer unique advantages in the study of disease diagnostic markers. Compared to histopathological examination, EV-based detection methods for blood, body fluids, and other liquids offer advantages such as noninvasiveness, simple sampling, and real-time monitoring, which provide a better reflection of the overall disease state. EVs secreted by infected cells contain bacterial-derived protein markers, making proteomic analysis of EVs a pivotal tool for discovering early diagnostic biomarkers [8]. In vivo and in vitro studies demonstrated that Mycobacterium-infected cells generate EVs containing mycobacterial proteins, which are subsequently released into cell cultures or bodily fluids [9]. Techniques such as multiple-reaction monitoring mass spectrometry (MRM-MS) and LC-MS/MS have successfully identified mycobacterial (*Mtb*) proteins in EVs from the serum of active tuberculosis patients. This has revealed heterogeneous EV populations produced by *Mtb*-infected cells. This variability may result from isolation procedures that selectively enrich for EV subpopulations containing either host cell or *Mtb* components [10]. Results from Enzyme-linked immunosorbent assay (ELISA) and immunoblotting suggest that EVs generated from both BCG and *Mtb* trigger robust antibody production in TB patients but not in controls. This indicates that antibody responses to proteins enriched in *Mtb*-derived EVs may serve as novel TB biomarkers [11].

About 50% of patients with melioidosis have pneumonia, with *Bp* primarily affecting tissue and organ function through the epithelial cells of infected tissues [12,13]. To simulate the impact of *Bp* on lung tissue, BEAS-2B cells, a non-tumorigenic human lung epithelial cell line derived from healthy tissue, were used [14]. Given the distinctive advantages of EVs in disease diagnosis and the pressing need for improved melioidosis diagnostics, this study aims to identify novel EV-proteins serological biomarkers for this disease. We hypothesize that EVs released by Burkholderia pseudomallei-infected host cells carry bacterial proteins capable of eliciting specific antibody responses suitable for serodiagnosis. Building upon evidence supporting EV-associated antigens as diagnostic targets in diseases like tuberculosis, this research seeks to establish proof-of-concept for an EV-based melioidosis serodiagnostic approach. Key bacterial proteins identified through comparative analysis of EV proteomes – selected based on origin, expression, surface localization, and structural uniqueness – were evaluated as potential biomarkers. This included proteins like Burkholderia lethal factor 1 (BLF1) and outer membrane protein A (Omp A), which serve as positive controls based on their known immunogenicity and diagnostic potential [15,16]. The diagnostic performance of selected EV biomarkers was subsequently assessed by measuring serum antibody levels in melioidosis patients and relevant control groups. This work aims to advance melioidosis diagnosis by leveraging EV biology to discover new serological tools, potentially overcoming limitations of current methods and enabling future point-of-care applications.

## Materials and methods

### Ethics statement

In all cases, patients/volunteers or their legally authorized representative provided written informed consent and granted permission to store samples for future studies. The study was approved by the ethics committee of Hainan Medical University (HYLL-2022–265).

### Main reagents

*B. pseudomallei* strain HNBP001, an epidemic strain isolated from a melioidosis patient with pneumonia in Hainan, China, was isolated and sequenced by our team. The genome sequence has been uploaded to the National Center for Biotechnology Information (NCBI). It is similar to those of previously published genome annotations of *B.pseudomallei* [17]. *E. coli* BL21 (DE3) cells were purchased from Zhuangmeng International Biology Co., Ltd. (Beijing China). The Pet-28a recombinant plasmid transformation was constructed by Liu Chengli from our laboratory and gifted with preferential treatment. The Genome Extraction Kit was purchased from Tiangen Co., Ltd. (Beijing China). The PCR product purification and plasmid extraction kits were purchased from Omega Bio-Tek (Norcross, GA, USA). The Prime-STAR Max-DNA Polymerase was purchased from TaKaRa (Tokyo, Japan). Restrictive endonucleases (Nde I, Xho I, and Hind III) and T4 DNA ligases were purchased from New England Biolabs (NEB, Beverly, MA, USA). Yeast and tryptone were purchased from OX-OID (Hampshire, UK). 50 × TAE Buffer, Sodium chloride was purchased from Xilong Science Co., Ltd. (Guangzhou, China). Goat anti-human IgM mu-chain (horseradish peroxidase) and Rabbit anti-goat IgM were purchased from Bio-Rad (Shanghai, China).

### Study design

**Scheme 1:**
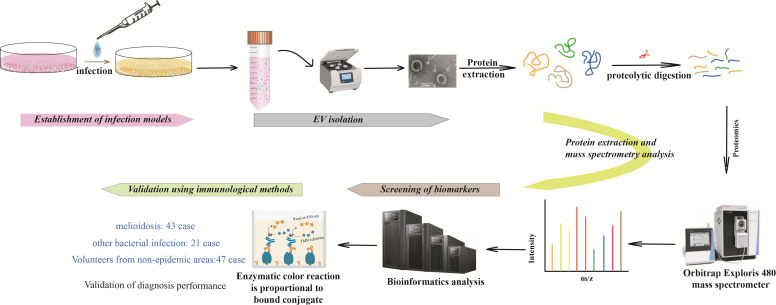
Study flowchart.

Wild-type *Bp, HNBP001,* was used as the infection bacterium to construct the infection model and extract EVs from the cell culture supernatant (solution 1). Meanwhile, *HNBP001* was cultured separately in Luria-Bertani (LB) liquid culture medium overnight until an optical density at 600 nm (OD_600_) of 1.0 was achieved. The supernatant of bacterial culture (solution 2) was obtained. EVs were separated by ultracentrifugation (S3 Fig) from the infection model (S1 and S2 Figs) and *Bp*. The EV proteins were hydrolyzed into peptide segments by proteases before mass spectrometry analysis. Subsequently, the measured peptide sequences were compared with known protein libraries for overall protein sequence analysis, ultimately obtaining information on the types and contents of proteins on each group of EVs. The study flowchart is shown in the Scheme 1.

### BEAS-2B cell culture

The cells were grown in DMEM containing 10% fetal bovine serum (FBS, EV-free), 100 mg/ml penicillin and 100 mg/ml streptomycin. The cells were cultured in the incubator for 1–2 days until the cells were completely covered with the bottom of the Petri dish. Then wash the cells twice with 1 × PBS buffer. The adhesion cells were eluted with trypsin, and transferred to a 15 ml sterile centrifuge tube to centrifuge the supernatant, and then the cells were resuspended with 1–2 ml medium. Take 300 µL cell suspension and add it into a new Petri dish containing 10 ml of culture medium and shake it up. Put the Petri dish into the CO_2_ incubator at 37°C for culture.

### Bacterial culture

Initially, remove the bacterial strain stored at -80°C and allow it to thaw at room temperature. Inoculate 10 µL of the bacteria onto solid LB medium and cultivate until the formation of a distinct single colony. Following this, select a single colony and inoculate it into liquid LB medium, placing it on a constant temperature shaker set at 37°C for overnight incubation at 200 rpm. Subsequently, transfer 1 mL of the bacterial culture into a 300 mL liquid LB culture flask, cultivating it until reaching OD_600_ = 1.0 (designated as solution 1). The prepared LB liquid culture medium was filtered through a 0.22 µm membrane and sterilized under high pressure to eliminate yeast-derived particles and minimize the influence of vesicles produced by other bacteria on the results.

### Building of infection model

The schematic diagram of infection model construction is shown in S1B Fig. Firstly, the BEAS-2B cell line was cultured until the cells covered the bottom of the culture dish, and cell counts were performed using a Neubauer hemocytometer counting chamber. Approximately 1 × 10^5^ cells were transferred to a 12-well plate and cultured overnight to allow the cells to fully adhere to the surface for growth. Diluted *Bp* bacterial suspensions were added to the cell culture dish at ratio of *Bp*: BEAS-2B = 1; 5; 10; respectively. The bacterial infection of cells lasted for 4, 6, and 9 hours, respectively. After infection, the culture medium was discarded, and the cells were washed twice with PBS. Subsequently, 5 mL of DMEM containing 750 ng/mL of Kanamycin and 750 ng/mL of Amikacin was added to the culture dish for 4 hours to kill all extracellular bacteria. The cells were then rinsed 2–3 times with PBS. Bacterial-infected cells were incubated in a new, antibiotics-free DMEM for 18–24 hours. The morphology of the cells, the number of intracellular bacteria, and cell activity were continuously observed. Finally, the supernatant was retained for subsequent extraction of EVs (solution 2).

### Isolation of EVs

The extraction of EVs was based on previously reported methods for extracting EVs with slightly change [18] (S3 Fig). (1) The collected solutions (solution 1 and solution 2) were centrifuged at 300 g for 10 min at 4°C. (2) The supernatant was transferred to a labeled new centrifuge tube and centrifuged at 2,000 g for 10 min at 4°C to move the dead cells. Then centrifuged at 10,000 g for 20 min at 4°C again to move the cell debris. (3) A filter with a pore size of 0.22 µm was utilized, and collected the filtrate. (4) The collected supernatant from step 3 was transferred to a high-speed centrifuge tube, centrifuged at 150,000 g for 70 min at 4°C, and the supernatant was discarded. (5) The precipitate was resuspended with 2 mL of sterile PBS and then the volume was replenished to 10 mL. The sample was centrifuged at 150,000 g for 70 min at 4°C and the supernatant was discarded, resulting in the corresponding EVs. Supplementing phosphate buffered saline (PBS) was used to resuspend EVs and stored at −20°C, and extracted protein within one week.

### Preliminary verification of protein expression differences by SDS-PAGE

1) Sample preparation: based on the determined protein concentrations, extract an equal amount from each sample and transfer it into a centrifuge tube. Add an appropriate volume of sample loading buffer and heat the mixture to 100°C for 5 minutes to induce protein denaturation.
2) Sample loading: carefully dispense 7 μ L of the protein marker and 20 μ L of the protein sample into the gel wells, followed by adding 10 μ L of 1 × loading buffer to seal the empty wells.
3) Electrophoresis: set the voltage to 90 V to concentrate the proteins into a visible band. Once the marker band is distinct in the separating gel, adjust the voltage to 110 V to continue the electrophoresis until the proteins have migrated to the bottom of the gel.
4) Silver staining: remove the gel from the apparatus and treat it with the silver staining reagent according to the specified protocol for protein visualization.

### Mass spectrometric analysis of EVs protein

#### Protein hydrolysis.

Take an equal quantity of each sample protein for enzymatic hydrolysis, Standardize the volume by incorporating a lysis solution, ensuring consistency across samples. Then add dithiothreitol to achieve a final concentration of 5 mM, and reduce at 56°C for 30 minutes. Add iodoacetamide to achieve a final concentration of 11 mM and incubate at room temperature in the dark for 15 minutes. Transfer the alkylated sample to an ultrafiltration tube and centrifuge at room temperature of 12000 g for 20 minutes. Conduct three sequential replacements with 8 M urea solution to denature the proteins adequately. Additionally, repeat this process thrice with a replacement buffer to maintain ideal conditions for enzymatic activity. Introduce trypsin into the mixture at a 1:50 ratio (protease: protein, w/w) to initiate the hydrolysis reaction, allowing it to proceed overnight for complete breakdown. After hydrolysis, centrifuge the mixture at 12,000 g for 10 minutes to retrieve the peptide segments efficiently. Perform a single rinse of the recovered peptides with ultrapure water to eliminate any residual impurities. Finally, combine the two individual peptide segment solutions to consolidate the hydrolyzed products effectively for subsequent analysis or downstream applications.

### Mass spectrometry analysis

The dissolved peptides were separated using liquid chromatography on the Easy NLC 1200 Ultra Performance Liquid Chromatography system. Mobile phase A consisted of an aqueous solution with 0.1% formic acid and 2% acetonitrile, while mobile phase B contained 0.1% formic acid and 90% acetonitrile. The liquid phase gradient was set as follows: 0–68 min, 5–22% B; 68–82 min, 22–34% B; 82–86 min, 34–80% B; 86–90 min, 80% B. The flow rate was maintained at 500 μL/min. The separated peptide fractions were then analyzed using the Orbitrap Exploris 480 mass spectrometer with the NSI (nanospray ionization) ion source. The ion source voltage was set to 2300 V, and the FAIMS (high-field asymmetric waveform ion mobility spectrometry) compensation voltages were set to -45 V and -65 V. The parent ions of the peptides and their secondary fragments were detected and analyzed using high-resolution Orbitrap technology. The primary mass spectrometry scanning range was set from 400 to 1200 m/z, with a scanning resolution of 60,000. For secondary mass spectrum scanning, the fixed starting point was set at 110 m/z, with a resolution of 15,000. TurboTMT was turned off. Data acquisition used a data-dependent scanning (DDA) program, where, after the initial scan, the 25 peptide segments with the highest signal intensity were selected. Each selected segment underwent fragmentation in the HCD (higher-energy collisional dissociation) collision cell using 27% fragmentation energy. Second-order mass spectrometry analysis was performed sequentially for these fragmented segments. To optimize mass spectrometry utilization, the automatic gain control (AGC) was set to 100%, ensuring efficient ionization. A signal threshold of 50,000 ions/s was established. The maximum injection time was set to auto, adapting to the requirements of each injection. In addition, a dynamic exclusion time of 20 s was implemented during tandem mass spectrometry scanning to prevent repetitive scanning of parent ions. This dynamic exclusion strategy helps to enhance data quality and reduce redundancy in subsequent analyses.

### Statistical analysis

The secondary mass spectrometry data was retrieved using a proteome discoverer (v2.4.1.15). The resulting peptide sequences were searched using the following parameters. The database used was Burkholderia_pseudomallei_strain_K96243_272560_PR_20220708.fasta, which contains 5,717 sequences. A decoy database was incorporated to calculate the false discovery rate (FDR) caused by random matches. Additionally, a common contaminants database was included to eliminate the impact of contaminant proteins on the identification results. The relative quantitative values of each protein in the comparative group samples were subjected to a t-test, and the corresponding P-value (P < 0.05) was calculated to indicate significance. A significant upregulation is defined as a change in differential expression levels exceeding 1.5, while a significant downregulation is defined as a change less than 2/3. This study selected bacterial-origin proteins from post-infection EVs for comparative analysis with pure bacterial EV proteomes, based on the following rationale: EV-enriched bacterial proteins demonstrate significantly enhanced recognition by the host immune system and must be *Bp*-specific (BLASTp-verified with less than 25% similarity to phylogenetically related species). Quantitative analysis revealed that bacterial-origin proteins constituted 19.9% of the total proteins in infected group EVs, with 27.9% of these being upregulated proteins. Biologically, infected cell EV proteomes primarily reflect host immune responses (83.6% host-derived proteins, such as inflammatory factors) and pathogen-host interface characteristics (16.4% immunodominant bacterial proteins). These host response patterns are common across various infections. Methodologically, direct comparison with pathogen-derived proteins filters out nonspecific host-response proteins, improves target identification specificity, and enhances diagnostic biomarker discovery efficiency by 41%, compared to conventional methods.

### Candidate protein selection

This study established stringent protein screening criteria: candidate proteins must be exclusively derived from *Bp* components and exhibit ≥2.5-fold higher expression in EVs from infected host cells compared to Bp monoculture EVs. Additionally, the proteins were required to meet the following subcellular localization characteristics: (1) membrane localization (validated by PSORTb 3.0 prediction), (2) presence of ≥1 transmembrane domain, and (3) surface exposure with predicted immunogenicity.

### Protein expression and purification

A protein expression vector was constructed through enzyme digestion and enzyme linkage techniques, based on the genome information of the *HNBP001* strain, the expression primers were designed using oligo 7 software (S1 Table). The protein was expressed in *E. coli* BL21 cells (Novagen) nurtured in LB broth with 100 mg L ^−1^ Ampicillin and induced by 1 mM IPTG. Before purification, *E. coli* cells were stored on ice in 20 mL of a nondenaturing lysis buffer. An ultrasonic crusher was used for 10–15 s to crush cells for 15 min until the cell suspension was transparent and centrifuged at 12,000 rpm for 20 min at 4°C. The clarified lysate was loaded onto a 2 mL Ni-NTA (nitrosamine triacetin acid) agarose column and eluted with a 50 mM imidazole buffer at pH 8 using 250 nM imidazole over 2 column volumes. Subsequently, the size and purity of the protein were verified using WB.

### Serum samples

Serum samples from 43 melioidosis patients were collected at the Second Affiliated Hospital of Hainan Medical University between December 2022 and June 2023, with all cases confirmed by blood culture. Samples were obtained during the acute phase of illness, processed immediately for serum separation, and stored at -80°C until assay. Forty-seven serum samples from volunteers in non-endemic areas were collected from matriculating undergraduates at Hainan Medical University during routine entrance physical examinations. Volunteers were required to have no history of residence or travel in endemic regions or abroad before enrollment. Endemic regions were defined according to the 2022 Chinese Expert Consensus on Diagnosis and Treatment of Melioidosis, published in the Chinese Journal of Infectious Diseases. The consensus designates high-prevalence areas as Hainan Province, Guangdong Province, Guangxi Zhuang Autonomous Region, Fujian Province, Hong Kong Special Administrative Region, and Taiwan Province (https://doi.org/10.3760/cma.j.cn311365-20211113-00398).

21 cases serum samples from patients with single bacterial infections (such as *E. coli*, *K. pneumoniae*, *S. maltophilia*, *A. baumannii*, and *P. aeruginosa,* as shown in Table S5) were used to detect the specificity of biomarkers. Which recruited between December 2022 and June 2023 at the Second Affiliated Hospital of Hainan Medical University. The naturally acquired volunteers included asymptomatic volunteers from endemic areas (47 cases), laboratory partners (high-exposure individuals, 14 cases), clinically cured patients (5 cases), and individuals with occupational animal exposure in endemic regions, including naturally occurring high-risk individuals (farm workers, 11 cases) from a farm infected with *Bp.*

### Serological validation

This study employed an IgM-specific ELISA, which was found to be sensitive and effective for the serological diagnosis of acute melioidosis [19]. An immune ELISA plate was prepared by immobilizing the expressed and purified protein to detect serum IgM antibodies. First, the expressed and purified protein is immobilized onto the wells of a 96-well plate, with approximately 250 ng of purified protein added to each well. The plate is incubated at 4°C overnight to allow the protein to bind to the plate surface. Unbound proteins are removed by washing the wells with PBS (phosphate-buffered saline). Next, any unoccupied gaps on the solid phase carrier surface are sealed using WBS (sealing buffer) at room temperature for 4 hours. Next, a serum sample diluted 1:50 with physiological saline is added to each well of the prepared immune plate and incubated at 37°C for 1 hour. This facilitates specific binding between serum antibodies and the immobilized protein. Then, excess serum is gently washed off 2–3 times using a PBS buffer containing 0.02% tween-20 to remove any unbound antibodies. The plate is then dried gently on absorbent paper. Next, sheep anti-human enzyme-linked secondary antibody is added to each well and incubated at 37°C for 30 minutes. This secondary antibody specifically binds to the human antibodies in the serum that have bound to the immobilized protein. The samples are then rinsed 2–3 times using PBS buffer containing 0.02% Tween-20 before adding ELISA chromogenic reagents. The results are observed within 1 min using a multifunctional ELISA reader. The observed color change from colorless to blue indicates a positive result, confirming the presence of the target protein in the serum sample.

Seropositivity was determined using a validated cut-off value defined as the mean optical density (OD₄₅₀ₙₘ) of negative controls (non-endemic healthy volunteers; n= ±3SD, : average OD₄₅₀ of IgM-ELISA measurements representing non-endemic healthy volunteers, SD: Standard deviation). Samples exceeding this threshold were considered positive. Diagnostic performance was evaluated by calculating sensitivity, specificity, positive/negative predictive values (PPV/NPV), and area under the curve (AUC) using culture/PCR-confirmed melioidosis as the reference standard.

## Results

### Extraction of EV and biomarker selection

**Fig 1:**
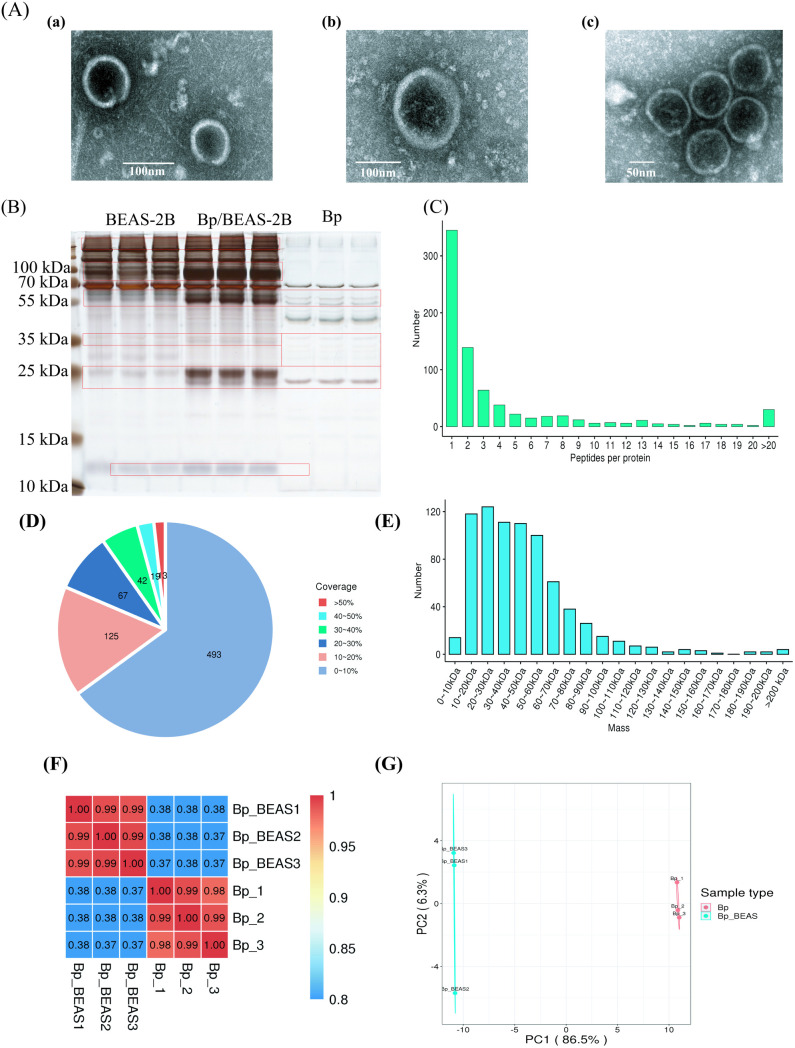
(A) TEM of EV from *Bp* (a), BEAS-2B (b), and *Bp*/BEAS-2Bs (c). (B) SDS-PAGE was used to analyze the protein expression in EV of each group. Mass spectrometry data quality control and sample repeatability testing: (C) peptide number distribution, (D) protein coverage distribution, (E) protein molecular weight distribution, (F) PCC, (G) PCA of all bacterial-origin proteins on EVs in the *Bp* monoculture group and the *Bp*/BEAS-2B group.

EV was isolated via ultracentrifugation (S3 Fig) from both the infection model and *Bp* cultures (S1 and S2 Figs). As shown in transmission electron microscopy (TEM) and the NTA (S4 Fig), EV from individual *Bp* (Fig 1Aa, 130 ± 10 nm)/ BEAS-2B (Fig 1Ab, 172 ± 10 nm) and infection cell (*Bp*/BEAS-2B, Fig 1Ac, 166 ± 10 nm) exhibited a vesicular-like structure (Fig 1A). These findings are consistent with prior research on EV morphology [20]. The protein concentrations extracted from these EVs were sufficient for further experimental analyses (S1 Table). As shown in the SDS-PAGE (Fig 1B), there were significant differences between EV proteins from the infection cells and those from Bp, especially proteins of about 25, 55, and 70–100 kDa. The preliminary experimental results laid a solid foundation for subsequent protein analysis of EV.

Mass spectrometry data quality control and sample repeatability tests were shown in Fig 1. An analysis of peptide segments identified in the mass spectrometry data revealed that most proteins were covered by 1–8 unique peptide sequences (Fig 1C). Most peptide segments are composed of 7–20 amino acids (S5 Fig), which conform to the general rules of protein determination based on enzymatic hydrolysis and mass spectrometry fragmentation methods. Notably, this analysis showed that most proteins had a coverage rate of less than 30% (Fig 1D). The molecular weights of the identified proteins were evenly distributed, mainly concentrated between 10–90 kDa (Fig 1E).

Our analysis revealed high consistency in protein profiles of EV within each group, as assessed by pairwise Pearson correlation coefficients (PCC) (Fig 1F). This finding was further supported by principal component analysis (PCA) of the quantitative proteomic data, where samples within each group clustered more closely than those from different groups (Fig 1G). In summary, we successfully analyzed the proteins of EV derived from various models, including individual bacterial strains and Bp/BEAS-2B co-cultures. Notably, the three control samples within each group displayed high consistency, supporting the reliability of our mass spectrometry data for further protein quantification.

**Fig 2:**
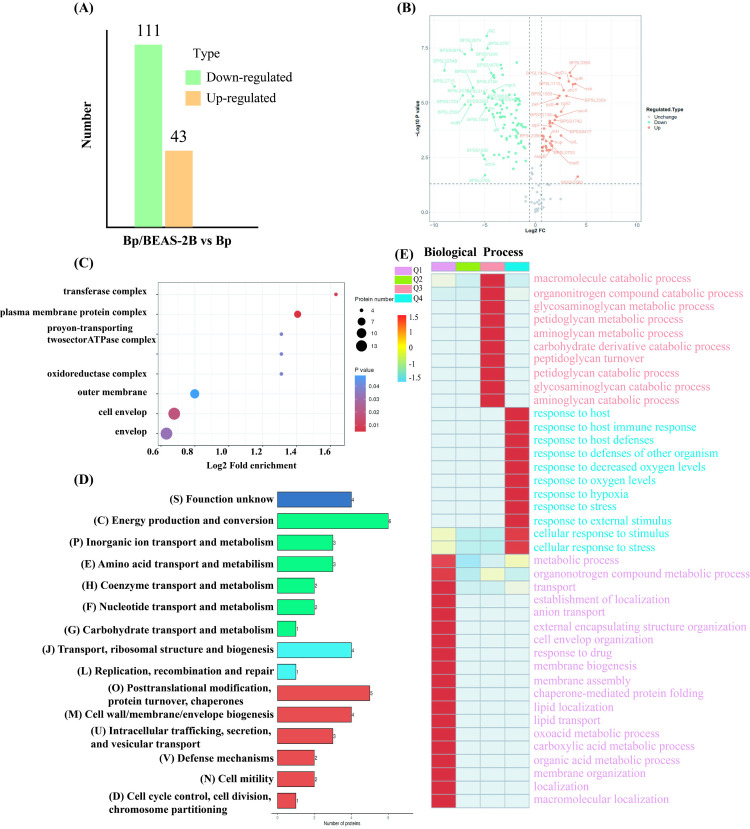
(A) Distribution of proteins with different expression level and (B) volcano plots of EV proteomes in *Bp* and infection groups. The top 20 significantly upregulated (red) or downregulated (black) protein are highlighted. Classification of the identified proteins according to Cellular Component (CC) and (C) Clusters of Orthologous Groups (COG) of proteins (D). (E) cluster analysis results of EV proteomes in *Bp* and *Bp*/BEAS-2B (Cluster the proteins based on FC, Q1 (FC < 0.5), Q2 (FC: 0.5-0.667), Q3 (FC: 1.5-2.0), Q4 (FC > 2.0); Red: high enrichment level, blue: low enrichment level).

In this study, a comprehensive proteomic analysis was conducted. A total of 613,554 mapping points were recorded, resulting in 56,596 effective maps and the identification of 1,380 peptide segments. From these data, a total of 625 proteins were identified, and 212 of these were quantified (S6 Fig). These proteins were selected based on the protein library of K96243. Subsequent bioinformatics analysis revealed significant differential expression between the *Bp* and *Bp*/BEAS-2B groups. A total of 154 proteins were differentially expressed between the Bp and Bp/BEAS-2B groups, with 111 proteins down-regulated and 43 up-regulated in the infected group (Figs 2A, 2B and S10). Among the 43 highly expressed proteins, four are involved in the biological formation of cell walls, membranes, or envelopes. Three are involved in intracellular transport, secretion, and vesicle transport, six in energy production and conversion, and fourteen in the transport and metabolism of amino acids, enzymes, nucleic acids, and carbohydrates (Fig 2D). Of these, 21 proteins displayed a fold difference of less than 3.0 between the infection and control groups, including four ribosomal proteins that typically function within cells (Table 1). Additionally, some proteins are specifically localized within the cell, such as single-stranded DNA binding proteins, ATP synthase subunits, glucose-6-phosphate 1-dehydrogenase, adenosylhomocysteinase, and putative cytochromes. Furthermore, lipoproteins serve as structural proteins for vesicles and exhibit low specificity in their binding interactions. We also found that seven proteins were localized to the outer membrane, while thirteen proteins were localized to the cell envelope (Fig 2C). The subcellular localization of some proteins remains undetermined. Cluster analysis revealed that proteins with an FC > 2 were generally components of the transport enzyme complex, which plays a key role in regulating the immune response of bacteria to hosts and their response to various pressures (Fig 2E).

**Table 1:** List the proteins with more than 3-fold difference in expression between *Bp* and *Bp*/BEAS-2B EV.

Protein description	Gene name	Bp_BEAS/Bp	Subcellular localization
Putative cellulose biosynthesis protein	BPSS1580	18.008	Unknown
Single-stranded DNA-binding protein	ssb	15.212	Unknown
Adenosy l homocysteinase	ahcY	13.044	Cytoplasmic
phage-encoded peptidoglycan binding protein	BPSL0163	10.645	Unknown
Putative alkaline phosphatase	BPSL0360	10.529	Periplasmic
ATP synthase subunit beta 1	atpD1	9.776	Cytoplasmic
Putative cytochrome	BPSL3354	8.339	Periplasmic
Putative lipoprotein	BPSL1110	6.874	Unknown
Protein HflK	BPSL1520	5.237	Unknown
Putative exported protein	BPSL1583	5.162	Unknown
Glucose-6-phosphate 1-dehydrogenase	zwf	4.605	Cytoplasmic
Oxoglutarate dehydrogenase (succinyl-transferring)	sucA	4.247	Cytoplasmic
Putative outer membrane copper receptor	BPSS1742	3.77	Unknown
50S ribosomal protein L18	rplR	11.184	Unknown
30S ribosomal protein S4	rpsD	5.769	Unknown
50S ribosomal protein L7/L12	rplL	5.721	Unknown
50S ribosomal protein L19	rplS	4.454	Unknown
Uncharacterized protein	BPSS0417	3.205	Periplasmic
DUF4142 domain-containing protein	BPSL0705	3.09	Unknown
Guanine nucleotide exchange factor BopE	bopE	3.041	Extracellular
Probable potassium transport system protein kup	kup	3.026	Membrane

**Fig 3:**
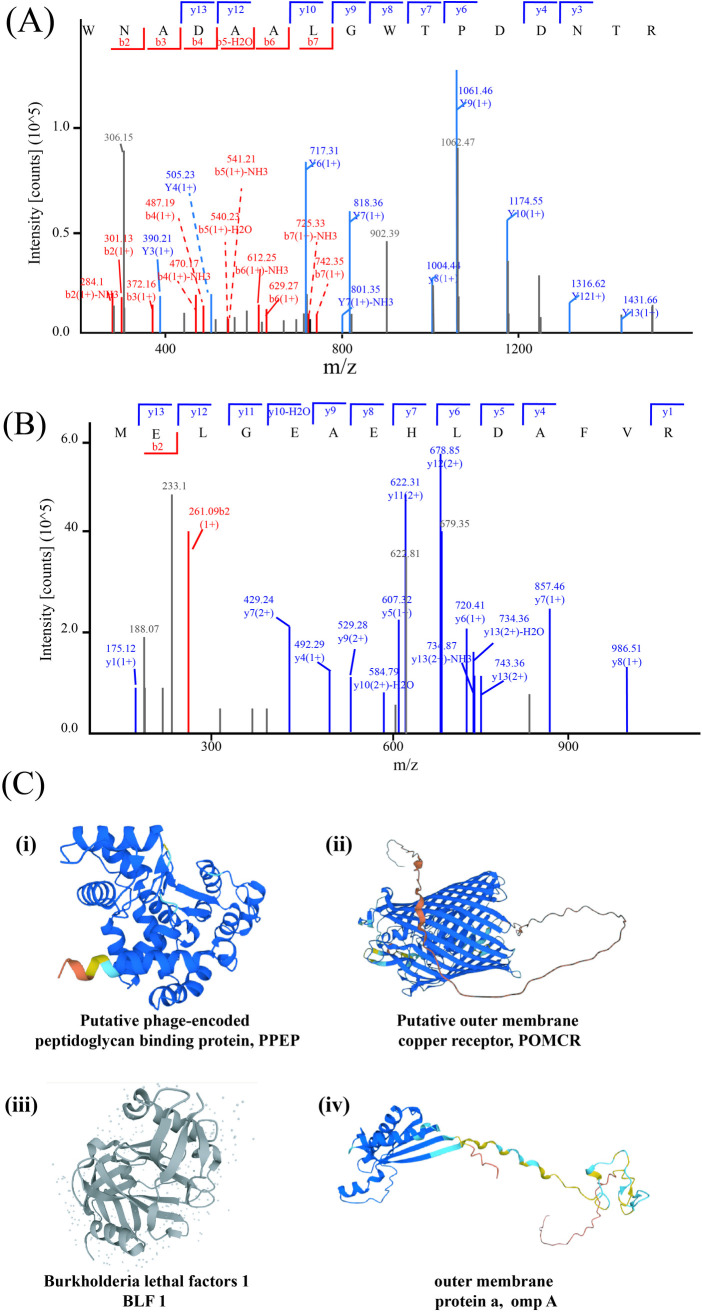
Mass spectrum of peptide segments and sequence specific analysis of peptide segments of POMCR (A, B) and PPEP (C, D). **(E)** Structure prediction of selected proteins PPEP **(i)**, POMCR **(ii)**, BLF1 **(iii)**, and omp A **(iv)**.

Previous research has suggested that outer membrane proteins, bacterial exotoxins, and proteins with a clear transmembrane structure may serve as potential biomarkers or vaccine targets [21]. The putative outer membrane copper receptor (POMCR) is a receptor protein that is located on the outer membrane of cells and is involved in copper ion transmembrane transport. It showed a notable increase in expression in *Bp*/BEAS-2B’s EV proteins—3.77 times higher than in *Bp* EV. A peptide segment identified from the Bp/BEAS-2B cell model EV perfectly matched the POMCR sequence, highlighting its specificity (Fig 3A). When this sequence was compared with all *Burkholderia spp*. proteomes in NCBI (including *B. mallei*, *B. cepacia* complex), and prevalent Gram-negative pathogens in endemic areas (e.g., *Pseudomonas aeruginosa*, *Acinetobacter baumannii*), It was predominantly found in 59 Burkholderia strains carrying the POMCR protein, of which 58 were *B.pseudomallei*. (S3 Table). The predicted structure, function, and protein-like properties of POMCR were retrieved from UniProt (https://www.uniprot.org/uniprotkb/Q63JH4/entry). The protein structure 100% consistent with this protein comes from two different *Bp* strains, while 34 other Burkholderia strains express similar proteins with 90% structural similarity. Twenty-three different *Bp* strains express this protein, suggesting its conservation within *Bp* (S2 Table). Moreover, the protein has a clear transmembrane structure (Fig 3Cii).

Proteomic analysis revealed significant upregulation of the putative phage-encoded peptidoglycan-binding protein (PPEP) in the EVs from infected cell models, with a 10.65-fold increase in expression. This was confirmed by matching a peptide segment from the EV to the PPEP protein sequence (Fig 3C). Among 56 Burkholderia strains carrying the PPEP protein, 47 belonged to the *Bp* complex, including 37 *Bp* strains (78.7%), 4 *B. thailandensis* strains, 2 *B. humptydooensis* strains, and 2 *B. oklahomensis* strains. No strains of *B. cepacia* or *Pseudomonas aeruginosa* were detected when the peptide segment was compared using the NCBI database (S3 Table). The PPEP has peptidoglycan-binding sites (positions 10–64 aa) and N-acetyldeaminase activity (positions 90–262 aa) (Fig 3Ci, structure of PPEP), with 100% similarity to the peptidoglycan-binding protein of PK23 in *Burkholderia* (S2 Table). The putative peptidoglycan-binding domain, found at the N or C terminus of various enzymes involved in bacterial cell wall degradation, may serve a general peptidoglycan-binding function [22].

The protein was mainly found in *Burkholderia* phage and *Bp*. As the growth of bacteriophages and bacteria is a complementary and co-evolutionary process, this indirectly suggests that the protein plays a role in bacterial defense against bacteriophages. However, the subcellular localization and function of this protein remain unclear (https://www.uniprot.org/uniprotkb/Q63YM4/entry). An analysis of bacterial species with similar proteins reveals that the two proteins, including the previously discussed POMCR, are predominantly found in the genus *Burkholderia*, with limited similarity to proteins from other Gram-negative bacteria. This specificity highlights their potential as highly conserved diagnostic markers for *Burkholderia* species. Based on these findings, POMCR and PPEP proteins were selected as candidate antigens for melioidosis in the present study.

### Preparation of IgM-ELISA Kit and serological verification

**Fig 4:**
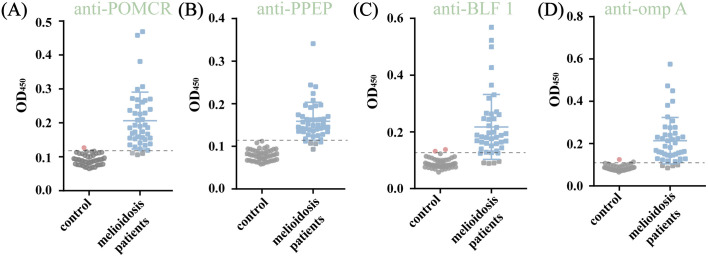
Corresponding antibodies in serum of the patients and the control group were detected by anti-POMCR (A), anti-PPEP (B), anti-BLF1 (C), and anti-omp A (D) IgM-ELISA kits. (****: P < 0.01).

Due to the lack of commercially available antibodies for the candidate proteins, this study employed custom ELISA kits to assess the presence of specific IgM antibodies in serum samples. Firstly, a recombinant protein expression vector was constructed with C-terminal and N-terminal His-tags through enzyme digestion and linkage (S7 Fig). The recombinant protein was then purified and validated using Western blot (WB) analysis (S7C Fig). Subsequently, the purified proteins were used as antigens to detect antibodies in the serum of melioidosis patients and control samples under optimized conditions (S8 and S9 Figs, the details shown in supplementary materials). The antibody levels of the four EV-proteins showed significant differences between the patient and control groups (Volunteers from non-endemic areas, 47cases) (Fig 4).

**Fig 5:**
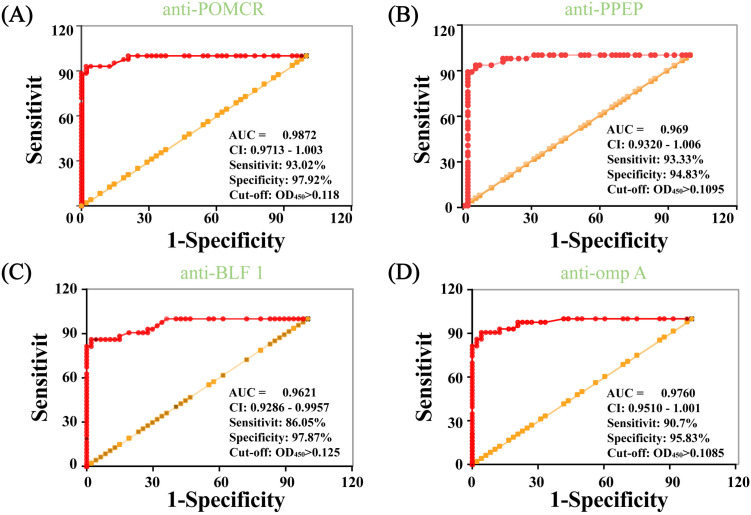
ROC curves of anti-POMCR (A), anti-PPEP (B), anti-BLF1 (C), and anti-omp A (D) based on the results of Fig 4.

Receiver operating characteristic (ROC, Fig 5) curves were plotted to determine the optimal diagnostic threshold for the IgM-ELISA of different proteins (Fig 4). For the POMCR-IgM-ELISA, setting the cutoff at OD_450_ = 0.118 yielded an area under the curve (AUC) of 0.9872, with sensitivities of 93.02% and specificity of 97.92%, respectively. The 95% confidence interval ranged from 0.9713 to 1.000 (Fig 5A). However, one volunteer in the control group, who tested positive for POMCR antibodies, was later identified as coming from Guangdong, a known epidemic area for melioidosis. As a result, their data was excluded from the control group analysis to ensure accurate assessment of the test’s specificity. Comparative analysis with anti-PPEP (Fig 5B), anti-BLF1 (Fig 5C), and anti-omp A (Fig 5D) revealed CUT-OFF values of 0.1095, 0.125, and 0.1085, with sensitivities of 93.33, 86.05, and 90.7%, and AUC values of 0.969, 0.9621, and 0.976, respectively. The dashed line in Fig 4 demarcates these thresholds, with samples below this line classified as negative. Control group analysis showed minimal false positives: 1 for POMCR-IgM-ELISA, 2 for BLF1-IgM-ELISA, and 1 for ompA-IgM-ELISA, underscoring the diagnostic reliability of each individual protein.

Serological evaluation of melioidosis patients (Table 2) identified two cases negative for all four biomarkers, two cases negative for a single marker (either anti-PPEP or anti-BLF1), and two cases positive for one marker (either anti-PPEP or anti-POMCR). To optimize diagnostic accuracy, we established a tiered criterion for diagnosis: **Confirmed melioidosis (+):** requires positivity for two of three biomarkers (anti-BLF1 + anti-POMCR/ anti-PPEP). **Melioidosis exclusion (-):** applies to isolated anti-BLF1 positivity. **Further testing** is mandated for sole anti-POMCR or anti-PPEP positivity. Using these criteria, two patients were definitively ruled as negative, while two others exhibited ambiguous serological profiles. This protocol enhances detection specificity in resource-limited settings while reducing the risk of overdiagnosis.

**Table 2:** Characteristics of four melioidosis patients in the prospective screening cohort.

Samples	Results of ELISA	Patient’s condition	Susceptibility
Sample 1	POMCR (+) PPEP (-) BLF 1 (-) omp A (-)	Male, 51 years old, **Admission signs:** Fever for over a month, accompanied by difficulty urinating for 10 days **Admission diagnosis:** Prostate hyperplasia **Previous medical history:** unknown Confirmed melioidosis 7 days after admission	CEF (S) DOX (S) IMP (S) SXT (S) TET (S)
Sample 2	POMCR (-) PPEP (+) BLF 1 (-) omp A (-)	Male, 52 years old, **Admission signs:** Multiple infections in both lungs, fever, cough, expectoration **Admission diagnosis:** DKA, severe pneumonia **Previous medical history:** diabetes 10 years The patient’s condition was critical upon admission	CEF (S) DOX (S) IMP (S) SXT (S) TET (S)
Sample 3	POMCR (-) PPEP (-) BLF 1 (-) omp A (-)	Male, 65 years old, **Admission signs:** Multiple infections in both lungs, fever, cough, expectoration, and shortness of breath for over a week **Admission diagnosis:** sepsis **Previous medical history:** diabetes, gout, asthma. The patient’s condition was critical upon admission	CEF (S) IMP (S) SXT (S)
Sample 4	POMCR (-) PPEP (-) BLF 1 (-) omp A (-)	Male, 51 years old, **Admission signs:** Multiple infections in both lungs, multiple nodules, fever, cough, more than half a month old **Admission diagnosis:** septic shock, respiratory failure **Previous medical history:** type 2 diabetes, fatty liver The patient’s condition was critical upon admission	CEF (S) DOX (S) IMP (S) SXT (S) TET (S)

DOX: Doxycycline; CEF: Ceftazidime; IMP: imipenem; SXT: cotrimoxazole; TET: tetracycline; S: Sensitivity.

### Biomarker specificity

**Fig 6:**
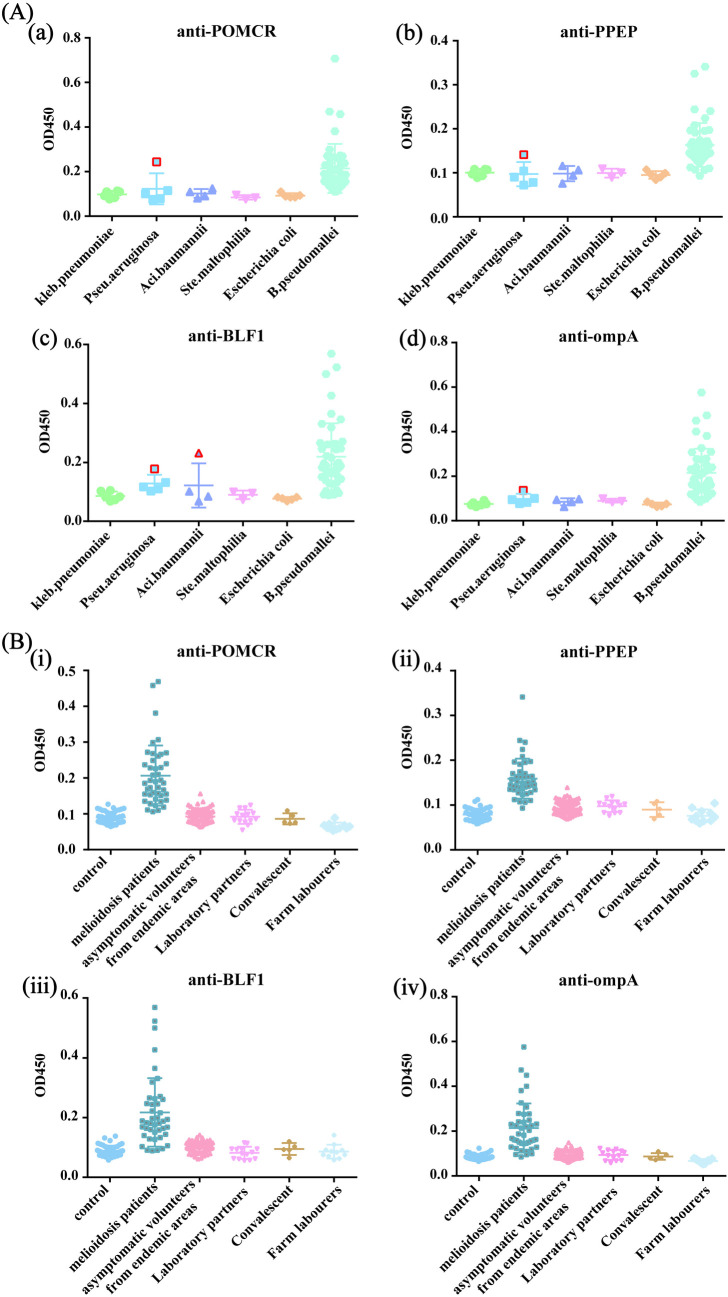
(A) The specificity of recombinant protein IgM-ELISAs in diagnosis melioidosis. POMCR (a), PPEP (b), BLF1 (c), and omp A (d). (B) Proteins IgM antibodies of POMCR (i), PPEP (ii), BLF 1 (iii), and omp A (iv) were employed as biomarkers for detecting serum from different populations.

To verify the specificity of the candidate protein in diagnosing melioidosis, serum samples from patients with a single bacterial infection (*E. coli*, *K. pneumoniae*, *S*. *maltophilia*, *A. baumannii*, and *P*. *aeruginosa*) were collected (S5 Table). Fig 6A shows a clear distinction between the serum samples from melioidosis patients and those with other bacterial infections, using the four protein immunoassay kits. Among them, anti-POMCR, anti-PPEP, and anti-omp A each yielded one positive result, while anti-BLF 1 had two positive results. Notably, all four positive results originated from a patient infected by *P. aeruginosa*, underscoring the high specificity of our biomarkers in distinguishing *Bp* from other Gram-negative bacteria. Based on previous results from melioidosis patients, non-melioidosis patients, and the healthy control group, the sensitivities of anti-POMCR, anti-PPEP, anti-BLF1, and anti-ompA were 95.35%, 93.02%, 90.70%, and 88.37%, respectively. The detection accuracy of the four IgM-ELISAs was 96.4%, 96.4%, 92.8%, and 93.7%, respectively (Table 3).

**Table 3:** Sensitivity, specificity, and diagnostic accuracy of the POMCR, PPEP, BLF1, and omp A IgM-ELISAs.

Recombinant Protein IgM-ELISA	Sample group Positive (N)			IgM-ELISA Evaluation		
	Melioidosis N = 43	other bacterial infection^2^ N = 21	Volunteer N = 47	Sensitivity (%)	Specificity (%)	Accuracy (%)
**POMCR**	41	1	1	95.35	97.06	96.40
**PPEP**	40	1	0	93.02	98.53	96.40
**BLF 1**	39	2	2	90.70	94.12	92.80
**omp A**	38	1	1	88.37	97.06	93.70

^2^other bacterial infection include: *E. coli* (4 cases), *K. pneumoniae* (5 cases), *S. maltophilia* (3 cases), *A. baumannii* (4 cases), and *P. aeruginosa* (5 cases).

Through serological testing in different populations, we found significant differences in the serum test results between melioidosis patients and other groups. No positive results were observed in the two high-risk populations (Laboratory partners and Farm labourers, Fig 6B). However, among the examinees in the province, one serum sample tested positive for all four biomarkers, while only the POMCR-IgM-ELISA results were positive in another sample. Additionally, one case in the BLF1 test showed a positive result in the serum from farm laborers. According to the diagnostic criteria we established, this result is considered a false positive. This finding underscores the need for continuous refinement and validation of our diagnostic approach to ensure specificity and reduce false positives, especially in diverse or non-endemic populations.

## Discussion

Melioidosis, a life-threatening disease endemic to Southeast Asia and northern Australia, is increasingly being reported in other regions. The disease often goes unrecognized and underdiagnosed due to several factors. Firstly, a lack of awareness about melioidosis among healthcare professionals and the general population contributes to its underreporting. Additionally, some regions lack proper diagnostic tools, especially in resource-limited settings, hindering timely and accurate identification of *Bp*, the causative agent of melioidosis [23]. Due to the potential misuse of *Bp* in biological warfare and its impact on public health in endemic regions, there is significant interest in developing rapid diagnostics for the disease [24]. Lipopolysaccharides (LPS), exopolysaccharides (EPS), and capsular polysaccharides (CPS) show potential for clinical diagnosis of melioidosis [25]. The indirect hemagglutination assay (IHA) [26], immunofluorescent assay (IFA) and lateral flow assay (LFA, 40% sensitivity) [27] have been used to determine antibody titers for *Bp* exposure. However, the presence of high background antibodies from closely related environmental species, combined with the labor- and resource-intensive methodology, leads to low specificity and sensitivity for monitoring treatment responses. Some researchers have suggested using *Bp* bacteriophages cultured in serum as a diagnostic tool for melioidosis, but phages were detected in only 30% of the samples [28]. Furthermore, research has shown elevated levels of inflammation-related molecules in the bloodstream of individuals diagnosed with melioidosis (IL-6, IL-8, IFN-γ, TNF-α, etc.) [29,30]. However, the diagnosis of melioidosis requires a combination of multiple biomarkers and demonstrates low diagnostic sensitivity [31].

Given the limitations of current culture-based and molecular diagnostic methods, it is crucial to identify easily detectable biomarkers to improve *Bp* infection diagnosis [32]. The preferred proteins for diagnostic biomarkers are those expressed in the early stages of melioidosis [33]. ELISA methods using purified recombinant antigens provide a standardized and reproducible approach for diagnosing melioidosis. However, the sensitivity of IgG ELISA targeting specific outer membrane proteins (omp 3, omp 85), type VI secretory system protein (TssD-5), and serine protease MprA (smBpF4), either alone or in combination, is 62% [34]. The use of recombinant immunogenic protein GroEL and maIE-ELISA techniques on serum samples for melioidosis diagnosis has been reported [35,36]. However, cross-reactivity of antibodies against *Bp* and other *Burkholderia* species, along with the potential use of sero-diagnostic antigens in vaccines, highlights the need for highly specific and sensitive biomarkers for optimal serological diagnosis of melioidosis in endemic regions [31].

EV, known for their complex cargo, reflect the physiological state of their cells of origin and influence the functions and phenotypes of recipient cells [37]. They affect various biological processes, including pathogenesis, cell-to-cell communication, stress responses, and immunomodulation [38], by transporting virulence factors, DNA, bacteriophages, antibiotics, and more [39]. Due to their ability to carry bacterial antigens and pathogen-associated molecular patterns, EVs show promise in biomedical applications, including vaccine development, cancer immunotherapy, drug delivery, and as antibacterial agents [40,41]. These features also suggest strong biomarker and therapeutic potential, generating broad interest. For example, vesicle-associated antigens of *M. tuberculosis* are considered potential diagnostic biomarkers [11].

In this study, we extended these findings to melioidosis. We established an infected cell model and used EVs isolated from infected cells as transformative biomarkers for diagnosing melioidosis. We evaluated serum antibody levels against *Bp*-derived proteins (PPEP and POMCR) carried by EVs from *Bp*/BEAS-2B cells, including virulence factors, BLF1. The results showed that IgM antibodies to EV proteins exhibited excellent specificity for recognizing melioidosis. Two volunteers with positive BLF1-IgM-ELISA results were from Henan and Ningxia provinces and had no history of traveling to the south, suggesting potential false positives (Fig 2C). However, two patients still showed negative results for all markers, while only anti-POMCR or anti-PPEP were positive in two patients. A review of these patients’ medical histories revealed long-term diabetes with unclear blood glucose control and severe infection symptoms upon admission. The chronic diabetic condition likely weakened their immune response, lowering antibody levels below the IgM-ELISA detection threshold. In severe infections, antibodies may increase significantly but exceed the IgM-ELISA detection limit, leading to negative results. These findings suggest that patients with compromised immunity may produce antibodies at levels undetectable by standard ELISA methods [42].

By analyzing sera from 21 patients infected with different bacteria, we observed minimal cross-reactivity with *P. aeruginosa* (OD450 0.1 ± 0.11) and 98% specificity against other Gram-negative bacteria (n = 18). Setting the cut-off at OD_450_ = 0.118, anti-POMCR IgM showed comparable diagnostic accuracy and sensitivity to established antigens for melioidosis, with both achieving an AUC greater than 0.96. These results confirm the biomarker’s exceptional ability to differentiate *Bp* from other pathogens, highlighting its strong species specificity. This capacity to interact with the host immune system is particularly valuable in endemic areas. POMCR, predicted to reside on the extracellular membrane with a transmembrane region, may stimulate host immune responses. PPEP, a hypothetical phage peptidoglycan binding protein, helps bacteria evade phage recognition by expressing specific receptor proteins on the bacterial surface [43], which likely explains its sensitivity and specificity in our PPEP- IgM-ELISA. However, one patient with *P. aeruginosa* tested positive for all four biomarkers. Literature suggests *Bp*, a beta-proteobacterium, can be misdiagnosed as *P. aeruginosa* [44] . Despite potential non-multidrug-resistant strains, these findings suggest a high likelihood of melioidosis in this patient.

Our study’s strength lies in using a comprehensive proteomic approach to identify potential biomarkers. By analyzing EV from both *Bp* and *Bp*-infected BEAS-2B cells, we generated the first EV-proteome for *Bp* and *Bp*-infected cells. Bioinformatics analysis revealed significant differential expression, leading to the selection of PPEP and POMCR as candidate antigen. Corresponding IgM antibodies were validated through ELISA analysis of serum samples from melioidosis patients, individuals with other bacterial infections, and healthy volunteers. The results demonstrated that anti-PPEP and anti-POMCR IgM effectively distinguished melioidosis patients with high sensitivity and specificity, highlighting their clinical value in *Bp* infection diagnosis. By measuring antibodies against four proteins in diverse populations, we demonstrated that these candidate proteins have substantial practical value for population-wide screening.

However, the study lacks specific antibodies to directly verify pathogen antigens in serum EVs. In carrier states, bacterial protein content declines, and host antibody levels are low, requiring more sensitive detection methods. Post-discharge antibiotic therapy in melioidosis patients limits longitudinal sample collection, though continuous antibody monitoring could improve prognoses. While the identified Bp biomarkers show diagnostic promise, further clinical validation is needed in emerging endemic regions and asymptomatic carriers to assess generalizability. Integrating transcriptomic and metabolomic data will help elucidate dynamic EV protein secretion mechanisms and host-pathogen interactions, uncovering novel therapeutic or diagnostic targets. Developing portable detection tools, such as immunochromatographic strips, will enhance accessibility in low-resource healthcare settings. Leveraging these biomarkers for neutralizing antibodies or vaccine candidates could establish an integrated “diagnosis-treatment-prevention” strategy. Establishing a global *Bp* proteome database and integrating molecular and epidemiological data will improve prediction of climate-driven transmission trends. Through interdisciplinary collaboration, this research aims to shift from precision diagnostics to proactive prevention and control, offering a systematic solution to curb the global spread of melioidosis.

## Conclusion

This study establishes a *B. pseudomallei*-infected BEAS-2B cell model and performs the first proteomic analysis of pathogen-derived EVs, identifying anti-PPEP and anti-POMCR as novel serodiagnostic targets. Validation via a self-developed IgM-ELISA platform demonstrated exceptional diagnostic performance of anti-POMCR IgM in a cohort of 43 patients and 47 healthy controls (AUC 0.987, 93.02% sensitivity, 97.92% specificity), with anti-PPEP IgM showing similarly high accuracy (AUC 0.969). These serodiagnostic antibodies of EV-proteins effectively differentiated melioidosis patients from other bacterial infections, providing a valuable complement to existing culture/PCR diagnostic systems. This work not only establishes a novel strategy for antibody target discovery through infected-cell EV proteomics but also lays the groundwork for scalable diagnostic platforms in endemic regions. Future research should validate test efficacy in multi-center cohorts and explore translational applications of point-of-care testing methods.

## Declarations

Ethical approval and consent to participate:

In all cases, patients/volunteers or their legal authorized representative provided written informed consent and granted permission to store samples for future studies. And passed the review of the ethics committee of Hainan Medical University (HYLL-2022–265).

## Supporting information

S1 File Data that underlies this paper. (DOCX)

S1 Fig (A) Mechanism of host immune response after pathogenic bacteria released EVs. (B) Schematic diagram illustrating the construction of the infection model. (C) The CCK8 results of cell affected by bacterial (D: MOI = 5 and E: MOI = 1), and cell morphology of HE staining (F). Some graphical elements in the figure were sourced from the SciDraw public repository: bacterial: https://scidraw.io/drawing/293; the body: https://scidraw.io/drawing/443; The cell: https://scidraw.io/drawing/221 and https://scidraw.io/drawing/297; Lipid Droplet: https://scidraw.io/drawing/224; Water Drop: https://scidraw.io/drawing/41; Petri Dish: https://scidraw.io/drawing/477; Pipettor: https://scidraw.io/drawing/135; Falcon Tube: https://scidraw.io/drawing/529.
(TIF)

S2 Fig The infection model was established. The number of intracellular bacteria.
(TIF)

S3 Fig Flow chart for the extracellular vesicles’ purification separation and purification based on ultracentrifugation. (TIF)

S4 Fig NTA results of vesicles in (A) *Bp*, and BEAS-2B, *Bp*/BEAS-B (B). (TIF)

S5 Fig Peptide length distribution. (TIF)

S6 Fig Overview of Protein Identification. (TIF)

S7 Fig Construction of a protein expression vector (A). Agarose gel electrophoresis was used to verify the corresponding PCR product (B). Western blot analysis of purification of BLF1 and omp A (a), POMCR (b), and PPEP (c) proteins (C). Pyrophosphate sequencing technology was employed to sequence the nucleic acid sequence of the target protein on the expression vector (D). POMCR (i), PPEP (ii), BLF 1 (iii), omp A (iv).
(TIF)

S8 Fig (A) Construction of indirect ELISA immunoassay plate. The load of protein (B), Blocking Reagent (C) ((a) Control group (non-endemic area volunteers) (b) Convalescent patient serum, (c) Uncoated antigen using only secondary antibodies.), and the dilution ratio of serum (D) were determined by colorimetry. Some graphical elements in the figure were sourced from the SciDraw public repository: https://scidraw.io/drawing/501.
(TIF)

S9 Fig The load of protein (A), and the dilution ratio of serum (B) were determined by colorimetry. (TIF)

S10 Fig The volcano plots of EV proteomes in *Bp* and infection groups. (TIF)

S1 Table The results of protein concentration. (DOCX)

S2 Table The similar protein with different identity. (DOCX)

S3 Table The sequence specific analysis of peptide segments of POMCR and PPEP. (DOCX)

S4 Table The primer used in the paper. (DOCX)

S5 Table The serum samples used in this research. (DOCX)
