# Supplementary material for: Proteomic-based identification of novel EV-derived protein antibodies biomarkers for melioidosis diagnosis
Source: PLoS Negl Trop Dis. 2025 Sep 24;19(9):e0013543. doi: 10.1371/journal.pntd.0013543 (PMC12459824; doi:10.1371/journal.pntd.0013543)
Supplement: S1 File — (DOCX) [file pntd.0013543.s001.docx]

**Supporting information**

**P****roteomic-based Identification of Novel EV-derived Protein Biomarkers for Melioidosis Diagnosis**

Nini Luo ^1, ¶^, Jun Tan ^1, ¶^, Xuemiao Li ^1, ¶^, Yanshuang Wang ^3^, Ting Zhang ^1^, Chen Chen ^1^, Lin Liu ^1^, Xinyi Song ^1^, Hua Pei ^3^, Bo Wang ^4^, Qi Li ^5^, Shen Tian ^1^, Nan Zhang ^1^, Wei Cheng ^2^, Qianfeng Xia ^1, *^

*^1^* *NHC Key Laboratory of Tropical Disease Control, School of Life Sciences and Medical Technology, Hainan Medical University, Haikou, Hainan, China*

*^2^ The Center for Clinical Molecular Medical Detection, The First Affiliated Hospital of Chongqing Medical University, Chongqing, P.R. China*

*^3^ The Second Affiliated Hospital of Hainan Medical University, Haikou, Hainan, China*

*^4^ Central Laboratory, Hainan General Hospital, Hainan Affiliated Hospital of Hainan Medical University, Haikou, Hainan, PR China.*

*^5^ The First Affiliated Hospital, Hainan Medical University, Haikou, Hainan, China*

^*^ xiaqianfeng@hainmc.edu.cn

¶ These authors contributed equally to this work.

**Results**

***Building of infection model***

After bacteria invade the human body through damaged skin or mucous membranes, EVs was secreted to target and attack immune or normal tissue cells, influencing immune responses by delivering bacterial-derived proteins. This aids in the clearance of bacteria and infected cells (S1A Fig) [16]. In cases where cells harbor bacteria without displaying active infection, these EVs communicate with other immune-related cells, enhancing their cytotoxic abilities and promoting the production of antibodies or initiating cellular immunity. This orchestration leads to the elimination of bacteria and carrier cells (S1 Fig).

To mimic the secretion state of cells in a carrier state, a meticulous bacterial infection cell model was established. By adjusting parameters such as infected time (IT), multiplicity of infection (MOI), and continuous culture time of *Bp*/BEAS-2B cells, a realistic simulation of the transition to a carrier state was achieved (S1B Fig). The intracellular bacterial load gradually increases (S1C and S2 Figs) and cell morphology was gradually affected with prolonged culture and infection time (S1F Fig) when the MOI = 1. As shown in S1F Fig, noticeable alterations in cell morphology were discernible at IT= 6 h; Cell death began to occur after IT was greater than 12 h, culminating in cell death at IT=18 h, while the cell activity was still greater than 0.8 (S1E Fig). At an MOI of 5 with a 4 h infection period, distinct morphological changes manifested after 18 h of culture (S1G Fig), but the cell activity was still greater than 0.8 (S1D Fig). The morphological changes and cell survival rate of cells at 6 h were like those at 4 h of infection. When the infection time is 9 h, the longer the culture continues, the greater the impact on cell morphology and activity. By synthesizing the comprehensive trends observed across various infection scenarios, the optimal conditions for cultivating the bacterial infection cell model were determined to involve an MOI of 5, a 6-hour infection period, and a subsequent 18-24 hours of continuous culture. These parameters set the stage for the subsequent detailed analysis of the cellular response dynamics in the context of bacterial infection.

***Protein expression***

A recombinant protein expression vector was constructed with both C-terminal and N-terminal His-tags using enzyme digestion and linkage methods (S6A Fig). Primers (S4 Table) were utilized to amplify the target protein on the developed plasmid, and the product was verified through agarose gel electrophoresis (S6B Fig) and pyrophosphate sequencing technology (S6D Fig). The analysis confirmed the successful construction of the vector without any mutation sites in the expressed protein, demonstrating a high degree of identity between the expressed protein and the protein in *Bp*. The recombinant vectors yielded proteins of the expected sizes: 80 kDa (POMCR), 29 kDa (PPEP), 23 kDa (BLF 1), and 27 kDa (omp A) (S6C Fig). The purity of the recombinant proteins was assessed via western blot analysis, revealing bands of consistent molecular weight with good homogeneity. The presence of a single protein band indicated high protein purity. Overall, the results confirmed the successful expression and purification of the four recombinant proteins.

***Self-made IgM-ELISA immuneplate***

A self-made indirect ELISA immunoassay plate was employed to detect the presence of specific antibodies in serum (S7A Fig). The amount of antigen coated on the experimental group (sample 1-4) and control group (sample 5-6) exhibited no notable impact on the subsequent color development process (S8A Fig). The analysis indicated an absence of significant differences in OD_450_ between the experimental group (patients) and the control group with varying protein coating quantities (S7B Fig). However, considering potential antigen loss during cleaning procedures, a consistent amount of 250 ng of protein coating was opted for all reaction wells.

Different blocking reagents, Western blotting sealing solution (WBS), 3% skimmed milk powder (3% SMP), 4% Fetal bovine serum albumin (4% BSA), were utilizied to prevent nonspecific binding on the immune-plate surface (S7C Fig). When using 4% BSA as blocking agent in the control group detection yielded a significantly higher OD_450_ compared to the other two reagents (P<0.05). An observable discrepancy in results emerged between WBS and 3% SMP when evaluating rehabilitation patient sera, with lower OD_450_ values observed with WBS blockade. When different blocking agents are used in the assay for unloaded antigen proteins, there is no difference in the experimental results (S7Cc Fig). The findings underscored the superior sealing efficacy achieved through WBS solution at ambient temperature.

The dilution ratio of serum was also explored in this article (1:50, 1:100, 1:200, 1:500). As the dilution ratio of serum increases, the color difference between positive group and the control group gradually decreases (S8B Fig), the signal-to-noise ratio (patient/control) decreases (S7D Fig). Therefore, the serum used for subsequent validation was diluted at 1:50.
