# Supplementary figures and images for: Proteomic-based identification of novel EV-derived protein antibodies biomarkers for melioidosis diagnosis
Source: PLoS Negl Trop Dis. 2025 Sep 24;19(9):e0013543. doi: 10.1371/journal.pntd.0013543 (PMC12459824; doi:10.1371/journal.pntd.0013543)

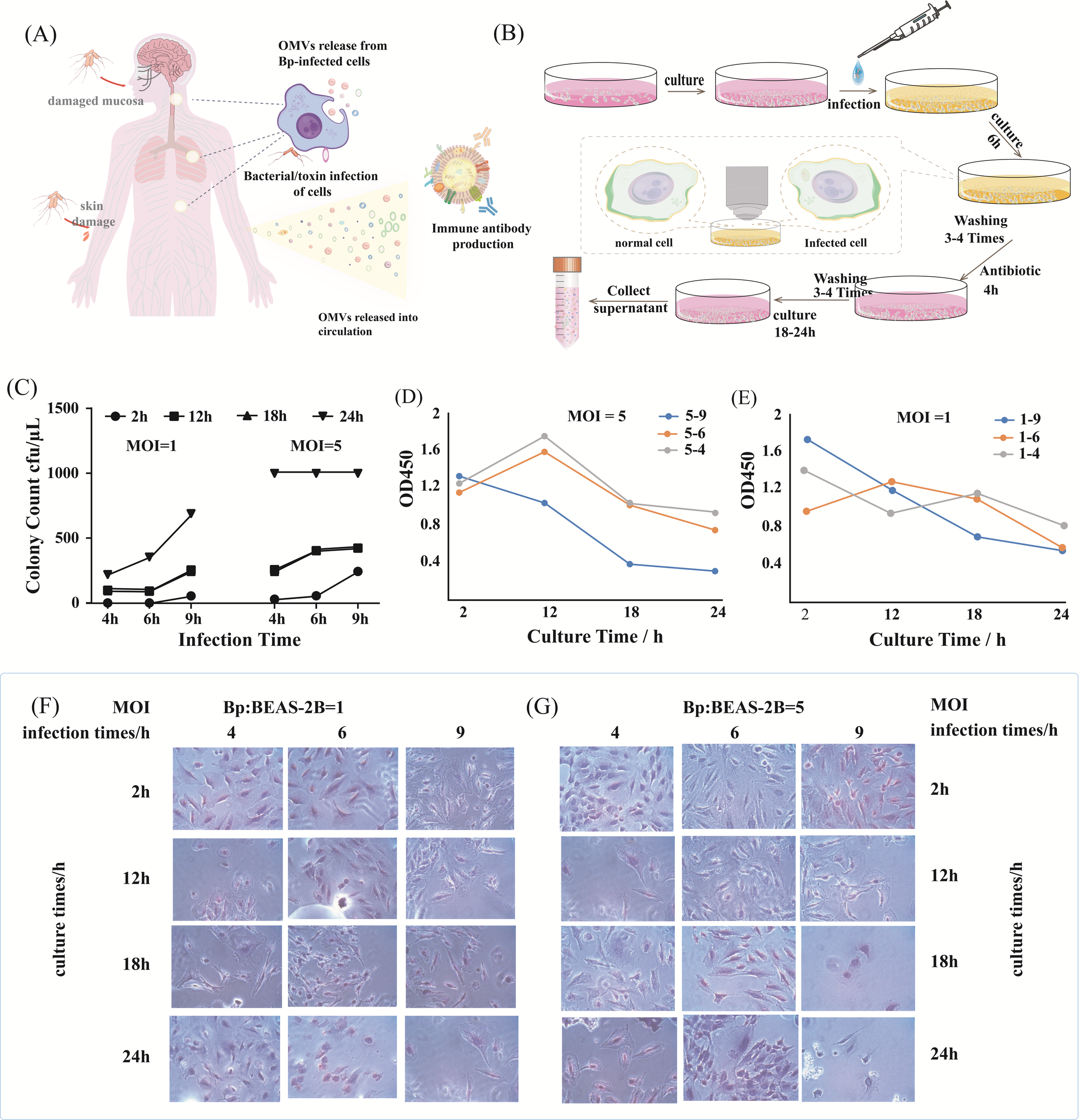

Supplement: S1 Fig — (B) Schematic diagram illustrating the construction of the infection model. (C) The CCK8 results of cell affected by bacterial (D: MOI = 5 and E: MOI = 1), and cell morphology of HE staining (F). Some graphical elements in the figure were sourced from the SciDraw public repository: bacterial: https://scidraw.io/drawing/293; the body: https://scidraw.io/drawing/443; The cell: https://scidraw.io/drawing/221 and https://scidraw.io/drawing/297; Lipid Droplet: https://scidraw.io/drawing/224; Water Drop: https://scidraw.io/drawing/41; Petri Dish: https://scidraw.io/drawing/477; Pipettor: https://scidraw.io/drawing/135; Falcon Tube: https://scidraw.io/drawing/529. (TIF) [file pntd.0013543.s002.tif]

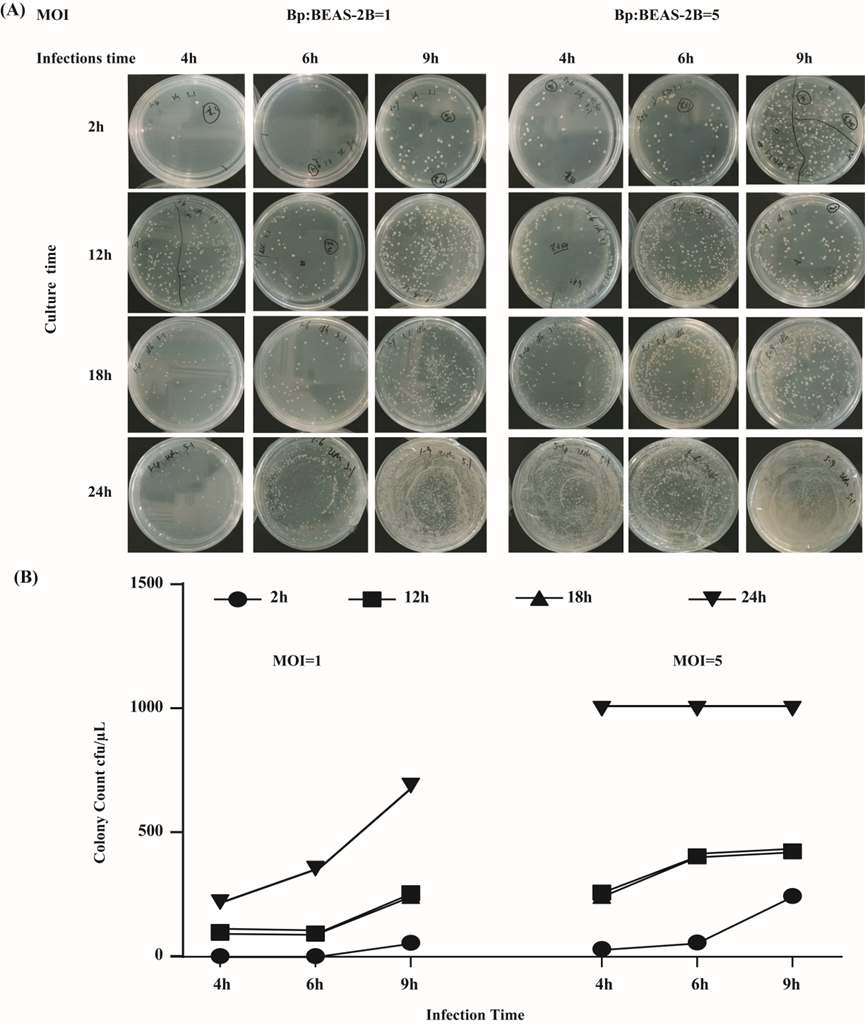

Supplement: S2 Fig — The number of intracellular bacteria. (TIF) [file pntd.0013543.s003.tif]

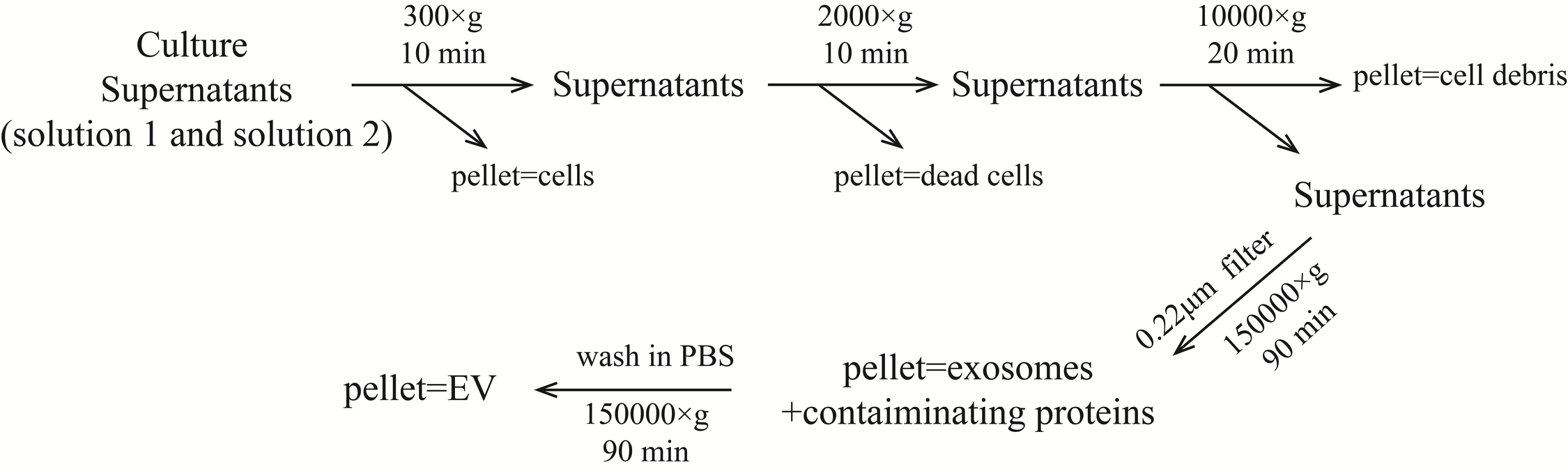

Supplement: S3 Fig — (TIF) [file pntd.0013543.s004.tif]

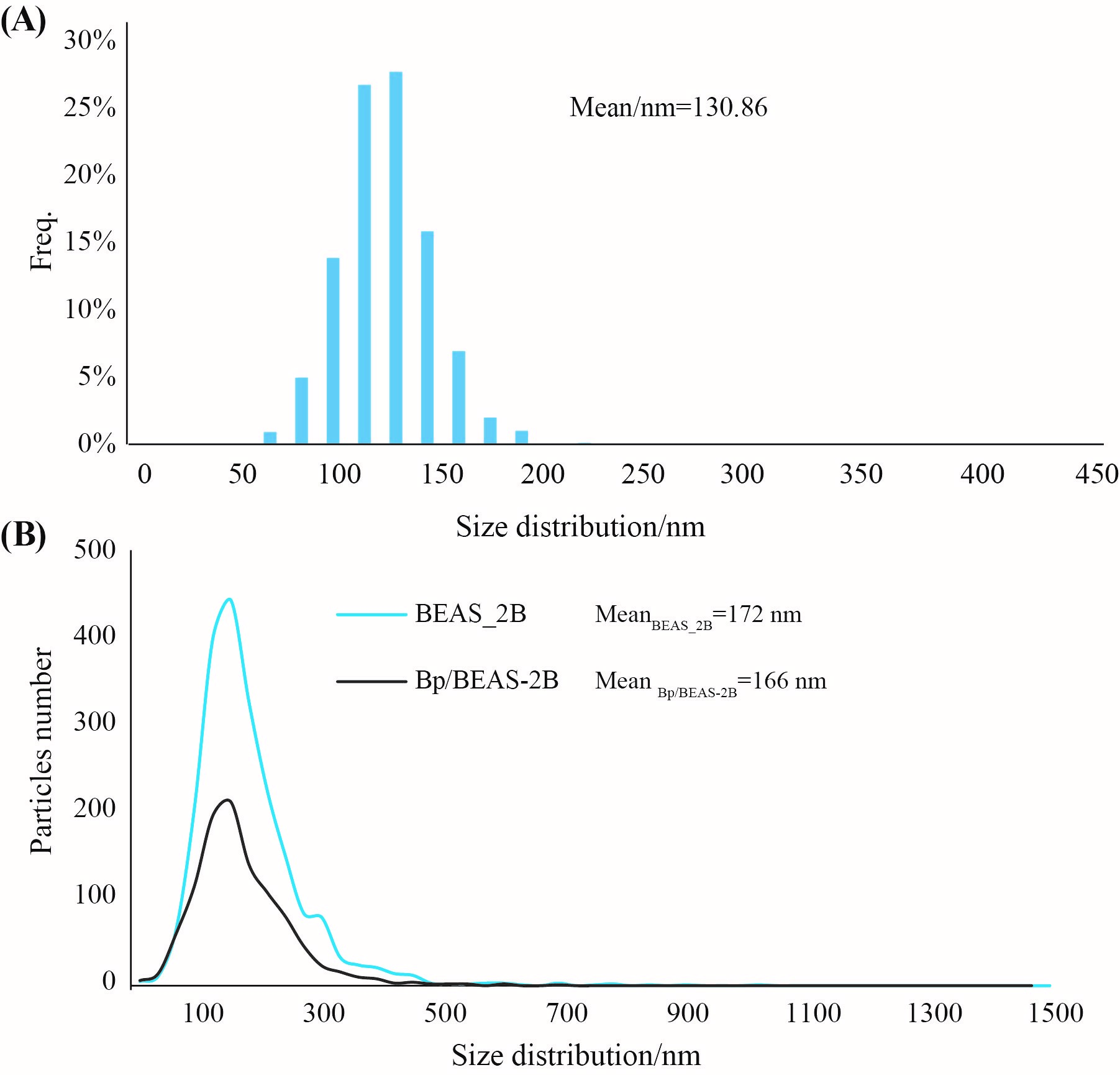

Supplement: S4 Fig — (TIF) [file pntd.0013543.s005.tif]

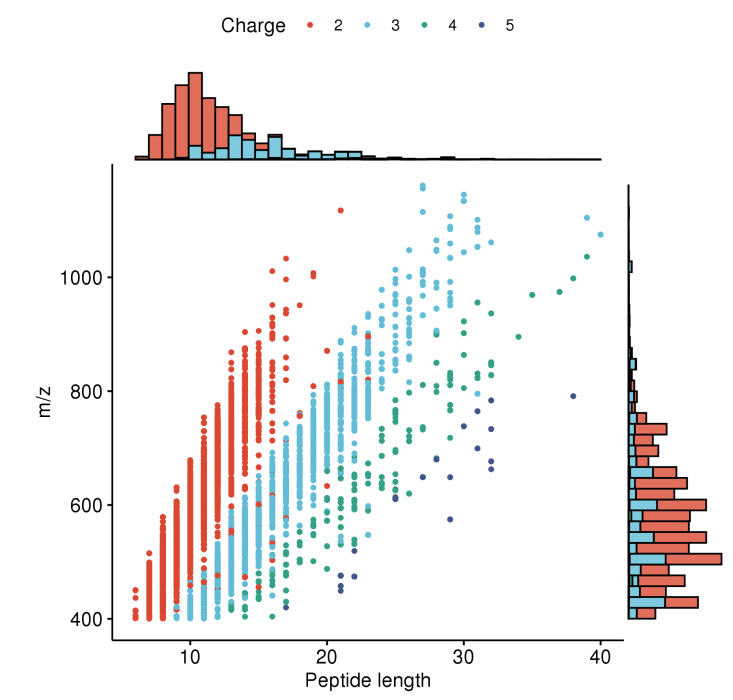

Supplement: S5 Fig — (TIF) [file pntd.0013543.s006.tif]

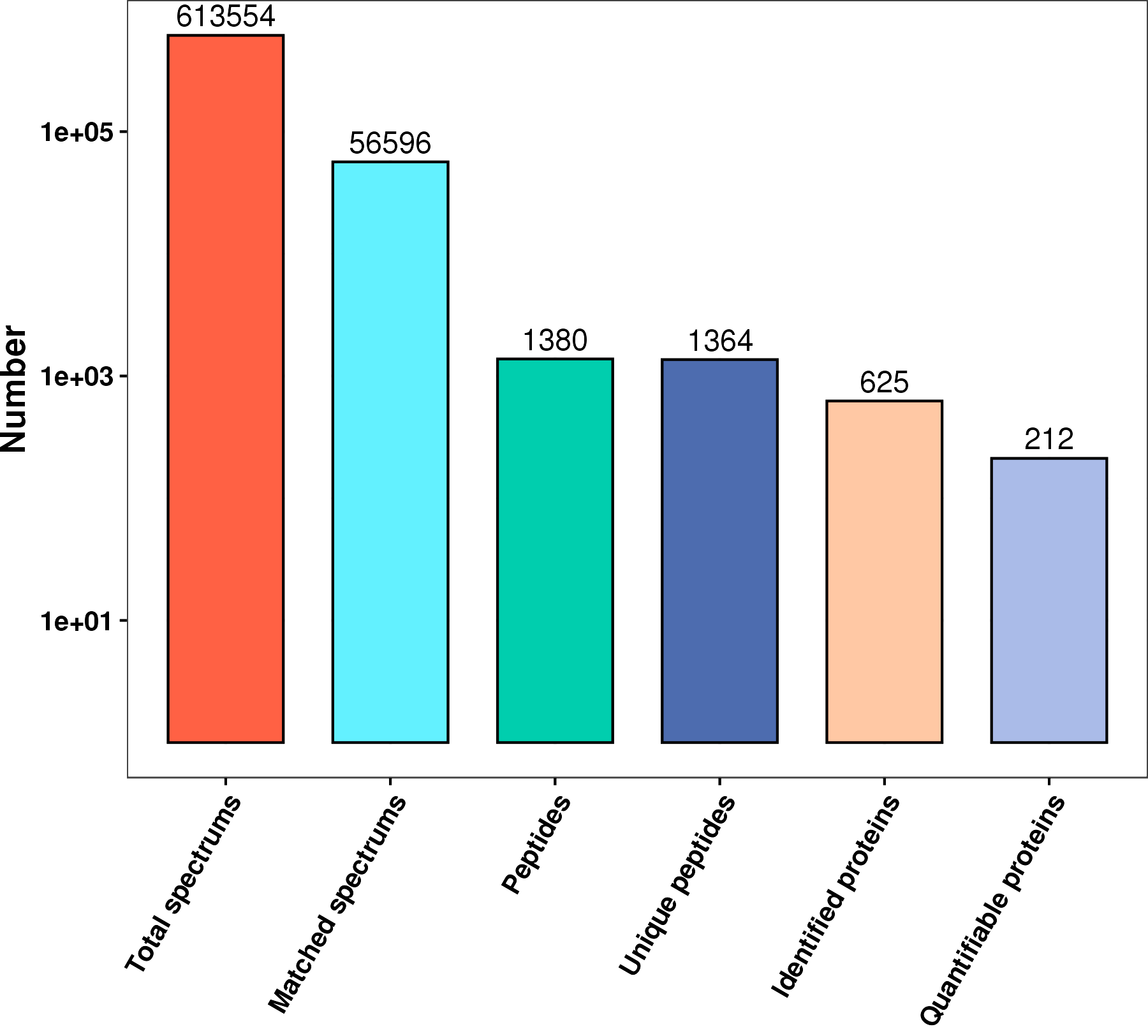

Supplement: S6 Fig — (TIF) [file pntd.0013543.s007.tif]

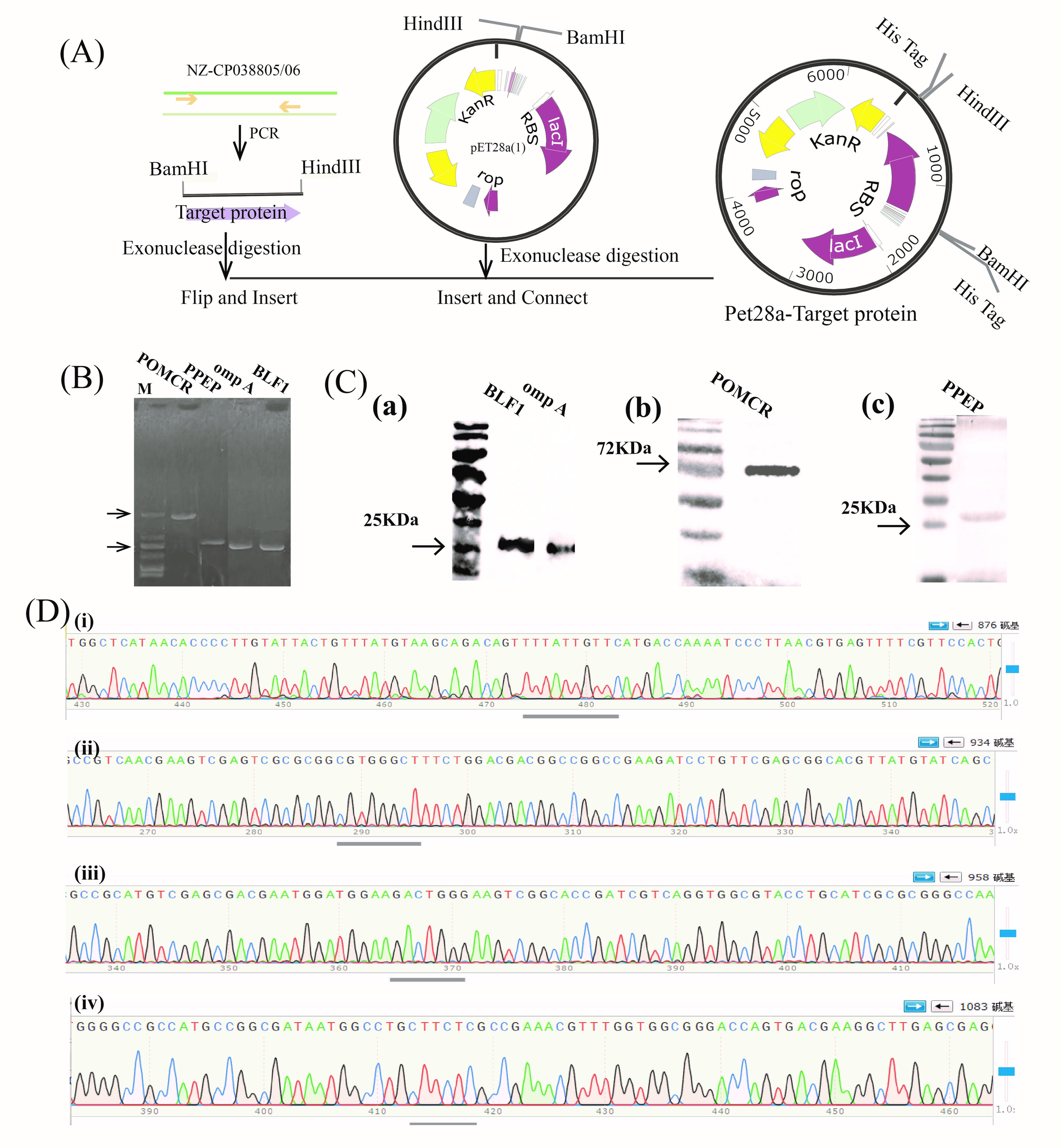

Supplement: S7 Fig — Agarose gel electrophoresis was used to verify the corresponding PCR product (B). Western blot analysis of purification of BLF1 and omp A (a), POMCR (b), and PPEP (c) proteins (C). Pyrophosphate sequencing technology was employed to sequence the nucleic acid sequence of the target protein on the expression vector (D). POMCR (i), PPEP (ii), BLF 1 (iii), omp A (iv). (TIF) [file pntd.0013543.s008.tif]

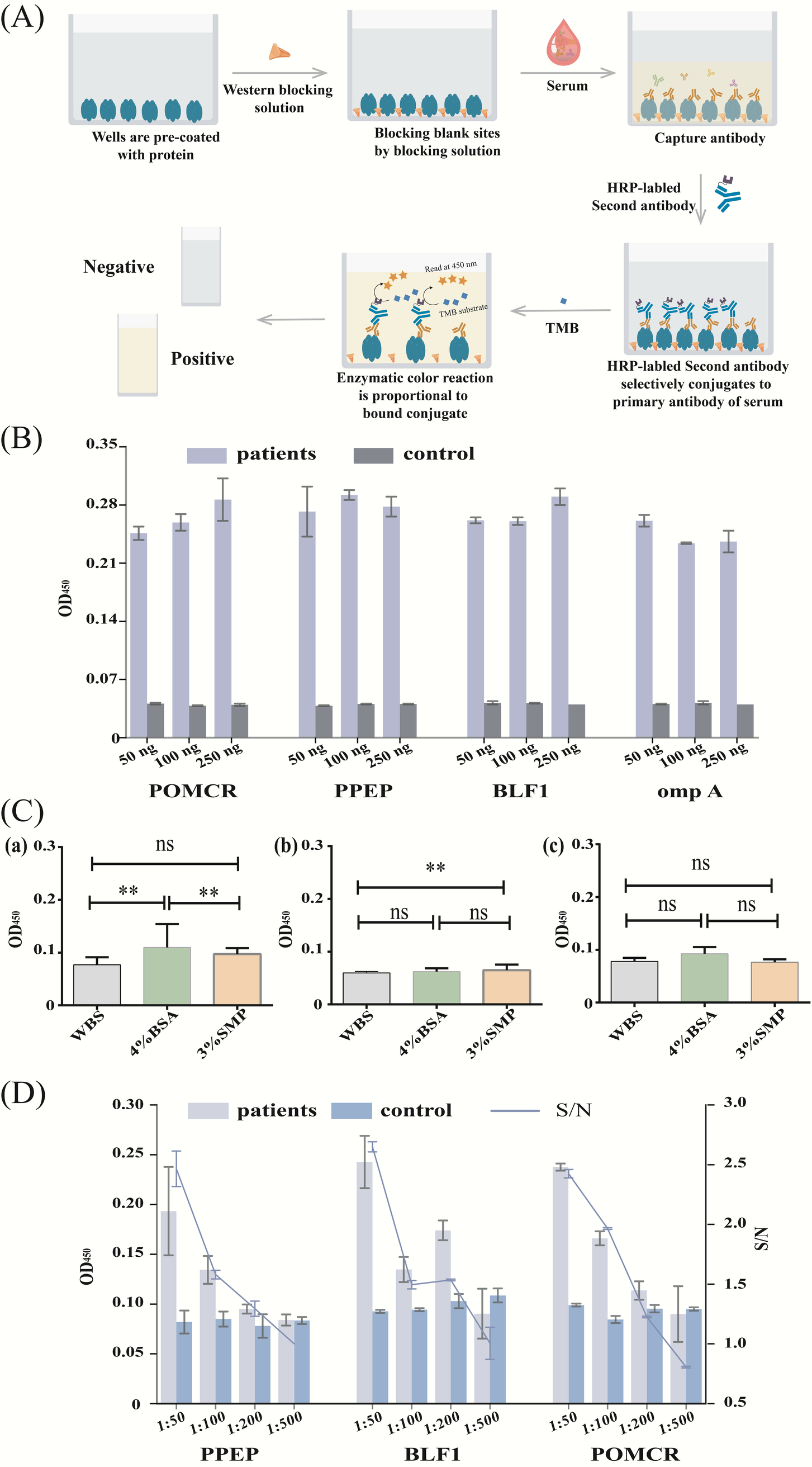

Supplement: S8 Fig — The load of protein (B), Blocking Reagent (C) ((a) Control group (non-endemic area volunteers) (b) Convalescent patient serum, (c) Uncoated antigen using only secondary antibodies.), and the dilution ratio of serum (D) were determined by colorimetry. Some graphical elements in the figure were sourced from the SciDraw public repository: https://scidraw.io/drawing/501. (TIF) [file pntd.0013543.s009.tif]

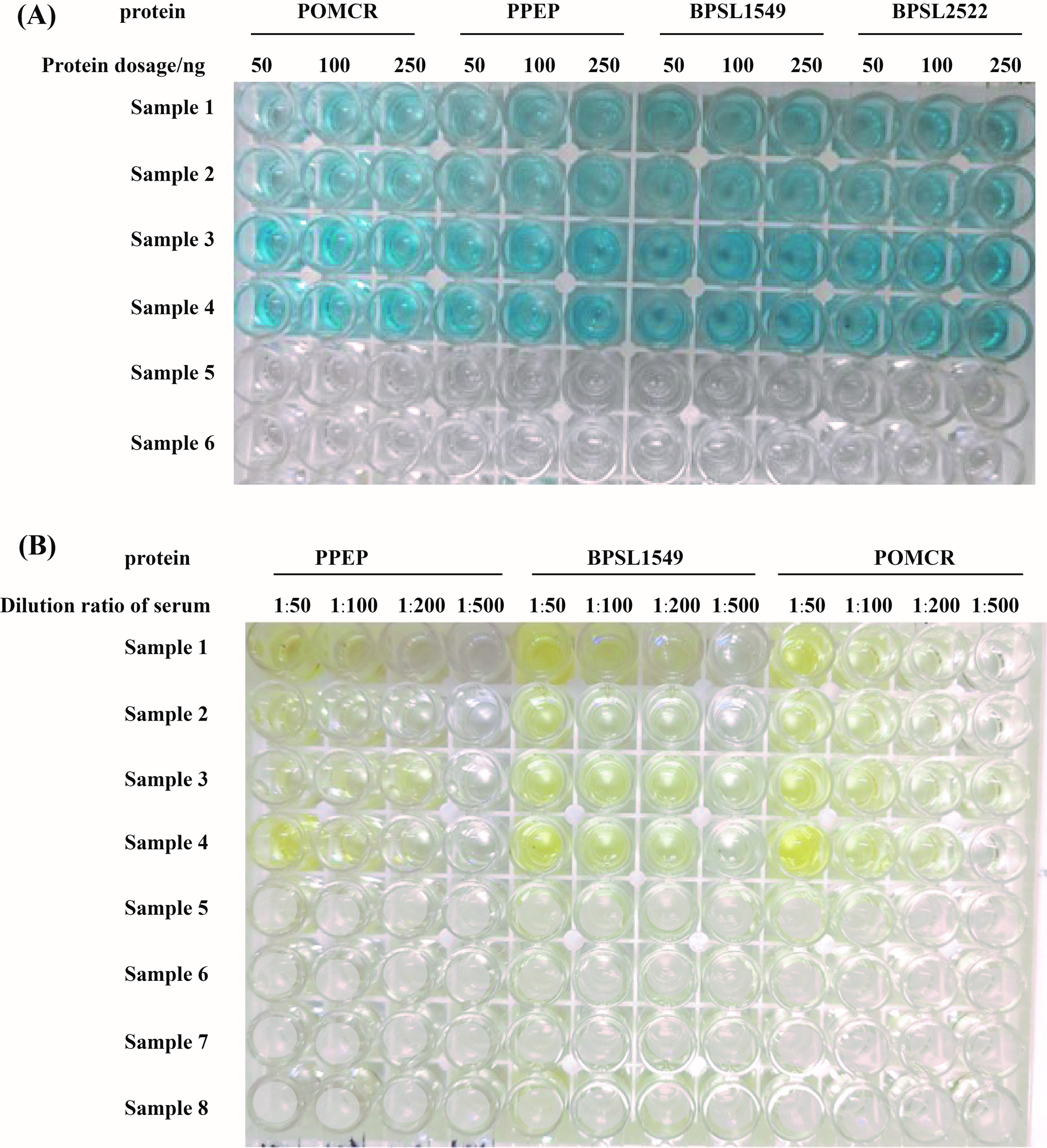

Supplement: S9 Fig — (TIF) [file pntd.0013543.s010.tif]

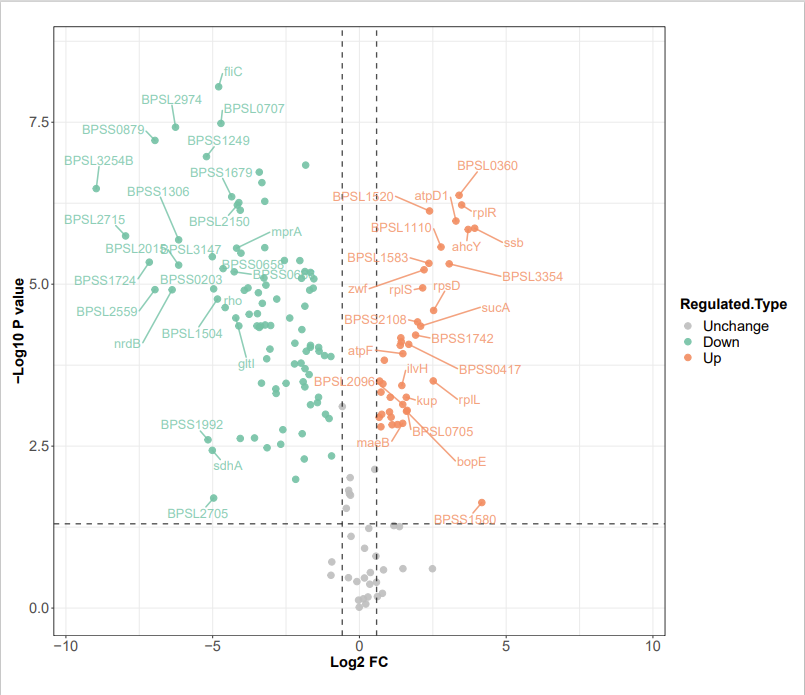

Supplement: S10 Fig — (TIF) [file pntd.0013543.s011.tif]
