# Supplementary material for: Proteomic-based identification of novel EV-derived protein antibodies biomarkers for melioidosis diagnosis
Source: PLoS Negl Trop Dis. 2025 Sep 24;19(9):e0013543. doi: 10.1371/journal.pntd.0013543 (PMC12459824; doi:10.1371/journal.pntd.0013543)
Supplement: S1 Table — (DOCX) [file pntd.0013543.s012.docx]

**S1 Table. The results of protein concentration**

| **Groups** | **Protein concentration (µg/µL)** | **Volume (µL)** | **Tatol protrin (µg)** |
| --- | --- | --- | --- |
| **BEAS_2B 1** | 2.65 | 130.0 | 344.8 |
| **BEAS_2B 2** | 2.64 | 130.0 | 343.2 |
| **BEAS_2B 2** | 2.67 | 130.0 | 347.1 |
| **Bp-BEAS-2B 1** | 6.97 | 260.0 | 1813.2 |
| **Bp-BEAS-2B 2** | 6.90 | 260.0 | 1794.0 |
| **Bp-BEAS-2B 3** | 6.98 | 260.0 | 1814.8 |
| **Bp 1** | 0.22 | 200.0 | 44.4 |
| **Bp 2** | 0.20 | 200.0 | 40.0 |
| **Bp 3** | 0.24 | 200.0 | 48.0 |
