# Supplementary material for: Proteomic-based identification of novel EV-derived protein antibodies biomarkers for melioidosis diagnosis
Source: PLoS Negl Trop Dis. 2025 Sep 24;19(9):e0013543. doi: 10.1371/journal.pntd.0013543 (PMC12459824; doi:10.1371/journal.pntd.0013543)
Supplement: S2 Table — (DOCX) [file pntd.0013543.s013.docx]

**S2 Table. The similar protein with different identity**

| **Protein** | **Protein name** | **Organism** | **Length** | **identity** |
| --- | --- | --- | --- | --- |
| **POMCR** | [TonB-dependent copper receptor](https://www.uniprot.org/uniprotkb/A0A0H3HVY2) | [B. pseudomallei (strain 1026b)](https://www.uniprot.org/taxonomy/884204) | 749 | 100% |
|  | [TonB-dependent copper receptor](https://www.uniprot.org/uniprotkb/A0A0H3HVY2) | [B. pseudomallei Pakistan 9](https://www.uniprot.org/taxonomy/595498) | 749 | 100% |
|  | [TonB-dependent copper receptor](https://www.uniprot.org/uniprotkb/A0A0H3HVY2) | [B. pseudomallei MSHR4000](https://www.uniprot.org/taxonomy/1435370) | 749 | 90% |
|  | [TonB-dependent receptor](https://www.uniprot.org/uniprotkb/A0A0H3HVY2) | [Burkholderia mayonis](https://www.uniprot.org/taxonomy/1385591) | 727 | 90% |
|  | [TonB-dependent copper receptor](https://www.uniprot.org/uniprotkb/A0A0H3HVY2) | [B. pseudomallei (strain 1106b)](https://www.uniprot.org/taxonomy/357347) | 749 | 90% |
|  | [TonB-dependent copper receptor](https://www.uniprot.org/uniprotkb/A0A0H3HVY2) | [B. pseudomallei 406e](https://www.uniprot.org/taxonomy/360118) | 749 | 90% |
|  | [TonB-dependent copper receptor](https://www.uniprot.org/uniprotkb/A0A0H3HVY2) | [B. pseudomallei (strain 1026b)](https://www.uniprot.org/taxonomy/884204) | 749 | 90% |
|  | [TonB-dependent receptor](https://www.uniprot.org/uniprotkb/A0A0H3HVY2) | [Burkholderia sp. MSMB617WGS](https://www.uniprot.org/taxonomy/1637831) | 750 | 90% |
|  | [TonB-dependent copper receptor](https://www.uniprot.org/uniprotkb/A0A0H3HVY2) | [Burkholderia humptydooensis](https://www.uniprot.org/taxonomy/430531) | 734 | 90% |
|  | [TonB-dependent receptor](https://www.uniprot.org/uniprotkb/A0A0H3HVY2) | [Burkholderia thailandensis](https://www.uniprot.org/taxonomy/57975) (strain ATCC 700388; DSM 13276; CCUG 48851; E264; CIP 1063-1) | 753 | 90% |
|  | [TonB-dependent copper receptor](https://www.uniprot.org/uniprotkb/A0A0H3HVY2) | [Burkholderia sp. Bp5365](https://www.uniprot.org/taxonomy/1740162) | 734 | 90% |
|  | [TonB-dependent copper receptor](https://www.uniprot.org/uniprotkb/A0A0H3HVY2) | [B. pseudomallei MSMB43](https://www.uniprot.org/taxonomy/360118) | 734 | 90% |
|  | [TonB-dependent copper receptor](https://www.uniprot.org/uniprotkb/A0A0H3HVY2) | [B. pseudomallei [(Pseudomonas pseudomallei)](https://www.uniprot.org/taxonomy/28450))](https://www.uniprot.org/taxonomy/884204) | 749 | 90% |
|  | [TonB-dependent receptor](https://www.uniprot.org/uniprotkb/A0A0H3HVY2) | [Burkholderia sp. ABCPW 14](https://www.uniprot.org/taxonomy/1637860) | 724 | 90% |
|  | [TonB-dependent copper receptor](https://www.uniprot.org/uniprotkb/A0A0H3HVY2) | [Burkholderia mallei (strain NCTC 10229)](https://www.uniprot.org/taxonomy/412022) | 745 | 90% |
|  | [TonB-dependent copper receptor](https://www.uniprot.org/uniprotkb/A0A0H3HVY2) | B. pseudomallei 305 | 745 | 90% |
|  | [TonB-dependent copper receptor](https://www.uniprot.org/uniprotkb/A0A0H3HVY2) | B. pseudomallei Pakistan 9 | 749 | 90% |
|  | [TonB-dependent receptor](https://www.uniprot.org/uniprotkb/A0A0H3HVY2) | [Burkholderia pseudomallei (strain 668)](https://www.uniprot.org/taxonomy/320373) | 745 | 90% |
|  | [TonB-dependent copper receptor](https://www.uniprot.org/uniprotkb/A0A0H3HVY2) | [Burkholderia mallei (Pseudomonas mallei; GB8 horse 4; ATCC 23344)](https://www.uniprot.org/taxonomy/13373) | 745 | 90% |
|  | [TonB-dependent copper receptor](https://www.uniprot.org/uniprotkb/A0A0H3HVY2) | [B. pseudomallei S13](https://www.uniprot.org/taxonomy/884204) | 749 | 90% |
|  | [TonB-dependent copper receptor](https://www.uniprot.org/uniprotkb/A0A0H3HVY2) | [Burkholderia mallei (strain NCTC 10247)](https://www.uniprot.org/taxonomy/320389) | 745 | 90% |
|  | [TonB-dependent receptor](https://www.uniprot.org/uniprotkb/A0A0H3HVY2) | [B. pseudomallei [(](https://www.uniprot.org/taxonomy/28450)strain1710a)](https://www.uniprot.org/taxonomy/884204) | 749 | 90% |
|  | [TonB-dependent copper receptor](https://www.uniprot.org/uniprotkb/A0A0H3HVY2) | [Burkholderia pseudomallei 1710a](https://www.uniprot.org/taxonomy/320371) | 749 | 90% |
|  | [TonB-dependent copper receptor](https://www.uniprot.org/uniprotkb/A0A0H3HVY2) | [B. pseudomallei [(](https://www.uniprot.org/taxonomy/28450)strainK96243, MSHR346, 1106a,)](https://www.uniprot.org/taxonomy/884204) | 749 | 90% |
|  | [TonB-dependent copper receptor](https://www.uniprot.org/uniprotkb/A0A0H3HVY2) | [B. pseudomallei [(](https://www.uniprot.org/taxonomy/28450)strain 576)](https://www.uniprot.org/taxonomy/884204) | 749 | 90% |
|  | [TonB-dependent copper receptor](https://www.uniprot.org/uniprotkb/A0A0H3HVY2) | [Burkholderia oklahomensis C6786](https://www.uniprot.org/taxonomy/441162) | 717 | 90% |
|  | [TonB-dependent receptor](https://www.uniprot.org/uniprotkb/A0A0H3HVY2) | [Burkholderia mayonis](https://www.uniprot.org/taxonomy/1385591) | 720 | 90% |
|  | **Extracellular copper receptor** | [B. pseudomallei (strain K96243; HNBP001)](https://www.uniprot.org/taxonomy/884204) | 749 | this work |
| **PPEP** | [Peptidoglycan binding protein](https://www.uniprot.org/uniprotkb/A0A8F3EA38) | [Burkholderia phage PK23](https://www.uniprot.org/taxonomy/2851080) | 270 | 100% |
|  | [Peptidoglycan binding protein](https://www.uniprot.org/uniprotkb/A0A8F3EA38) | [Burkholderia sp. Bp5365](https://www.uniprot.org/taxonomy/1740162) | 270 | 90% |
|  | [Peptidoglycan binding protein](https://www.uniprot.org/uniprotkb/A0A8F3EA38) | [Burkholderia phage PK23](https://www.uniprot.org/taxonomy/2851080) | 270 | 90% |
|  | [Putative peptidoglycan binding domain](https://www.uniprot.org/uniprotkb/B1H6A7) | [Burkholderia pseudomallei S13](https://www.uniprot.org/taxonomy/320374) | 270 | 90% |
|  | [Putative peptidoglycan binding domain protein](https://www.uniprot.org/uniprotkb/A0A0F6LCQ6) | [Burkholderia pseudomallei MSHR4000](https://www.uniprot.org/taxonomy/1435370) | 270 | 90% |
|  | [Putative peptidoglycan binding domain protein](https://www.uniprot.org/uniprotkb/A0A0F6LCQ6) | [Burkholderia pseudomallei (Pseudomonas pseudomallei)](https://www.uniprot.org/taxonomy/28450) | 270 | 90% |
|  | [Putative peptidoglycan binding domain protein](https://www.uniprot.org/uniprotkb/A0A0F6LCQ6) | [Burkholderia phage BEK](https://www.uniprot.org/taxonomy/1514988) | 270 | 90% |
|  | [Gp28-like protein](https://www.uniprot.org/uniprotkb/Q45YF3) | [Burkholderia phage phiE52237](https://www.uniprot.org/taxonomy/2681592); phiE094; phiX216 | 270 | 90% |
|  | [Bacteriophage-acquired protein](https://www.uniprot.org/uniprotkb/Q63LD8) | [Burkholderia pseudomallei (strain K96243)](https://www.uniprot.org/taxonomy/272560) | 270 | 90% |
|  | [Putative phage-encoded peptidoglycan bindingprotein](https://www.uniprot.org/uniprotkb/A0A0H3HFW3) | [Burkholderia pseudomallei (strain 1026b)](https://www.uniprot.org/taxonomy/884204) | 270 | 90% |
|  | **N-acetylmuramidase family protein** | [Burkholderia humptydooensis](https://www.uniprot.org/taxonomy/430531) | 270 | 90% |
|  | [DUF3380 domain-containing protein](https://www.uniprot.org/uniprotkb/A0A808PWZ2) | [Burkholderia thailandensis](https://www.uniprot.org/taxonomy/57975)(MSMB43) | 270 | 90% |
|  | **Phage-encoded peptidoglycan binding protein** | [B. pseudomallei (strain K96243; HNBP001)](https://www.uniprot.org/taxonomy/884204) | 270 | this work |
| **BLF1** | Uncharacterized protein | Burkholderia pseudomallei(strain 668) | 211 | 100% |
|  | Uncharacterized protein | Burkholderia mallei GB8horse4 | 144 | 100% |
|  | Uncharacterized protein | Burkholderia pseudomallei(strain1026b) | 211 | 100% |
|  | Uncharacterized protein | Burkholderia pseudomallei  (Pseudomonas pseudomallei) | 211 | 100% |
|  | Uncharacterized protein | Burkholderia pseudomallei305 | 211 | 100% |
|  | Uncharacterized protein | Burkholderia pseudomallei S13; K96243; MSHR4000; MSHR346 | 211 | 100% |
|  | Uncharacterized protein | Burkholderia pseudomallei576 | 211 | 100% |
|  | Uncharacterized protein | Burkholderia pseudomallei Pakistan9 | 211 | 100% |
|  | Uncharacterized protein | Burkholderia mallei(Pseudomonas mallei) | 132 | 100% |
|  | Uncharacterized protein | Burkholderia pseudomallei (strain 1106a; 1106b; 406e) | 211 | 100% |
|  | Uncharacterized protein | B. pseudomallei (strain668) | 211 | 90% |
|  | Uncharacterized protein | Burkholderia mallei GB8horse4 | 144 | 90% |
|  | Uncharacterized protein | Burkholderia pseudomallei (strain1026b) | 211 | 90% |
|  | Uncharacterized protein | Burkholderia pseudomallei305 | 211 | 90% |
|  | Burkholderia lethal factor 1 (BLF1) | [B. pseudomallei (strain K96243; HNBP001)](https://www.uniprot.org/taxonomy/884204) | 211 | this work |
