# Supplementary material for: Proteomic-based identification of novel EV-derived protein antibodies biomarkers for melioidosis diagnosis
Source: PLoS Negl Trop Dis. 2025 Sep 24;19(9):e0013543. doi: 10.1371/journal.pntd.0013543 (PMC12459824; doi:10.1371/journal.pntd.0013543)
Supplement: S3 Table — (DOCX) [file pntd.0013543.s014.docx]

**S3 Table. The sequence specific analysis of peptide segments of POMCR and PPEP**

|  | number of Organisms | | |
| --- | --- | --- | --- |
|  | PPEP | POMCR |  |
| Burkholderiaceae | 56 | 60 |  |
| pseudomallei group | 47 | 59 |  |
| *B. pseudomallei* | 37 (78.7%) | 58 (98.3%) |  |
| *B.oklahomensis* | 2 (4.3%) | / |  |
| *B.humptydooensis* | 2 (4.3%) | / |  |
| *B. thailandensis* | 4 (8.5%) | / |  |
| The data were obtained by comparing the measured peptide sequences with all protein peptide sequences in the NCBI database. | | | |
