# Supplementary material for: Proteomic-based identification of novel EV-derived protein antibodies biomarkers for melioidosis diagnosis
Source: PLoS Negl Trop Dis. 2025 Sep 24;19(9):e0013543. doi: 10.1371/journal.pntd.0013543 (PMC12459824; doi:10.1371/journal.pntd.0013543)
Supplement: S4 Table — (DOCX) [file pntd.0013543.s015.docx]

**S4 Table. The primer used in the paper**

| **protein** | **primer** | **sequence** | **Length (nt)** |
| --- | --- | --- | --- |
| **POMCR** | POMCR-F | AAAACTCGAGACGTCAATAGCGCGCGTTGAGCGTG | 35 |
|  | POMCR-R | TTTTCATATGCCGATGAAGTCGCGTTCCGACGAG | 34 |
| **PPEP** | PPEP-F | TTTTCTCGAGAGGTTCATGCGGCCGCCTTGCCCGT | 35 |
|  | PPEP-R | TTTTCATATGTGAACATCCTTCGATTCAACGACCA | 35 |
| **BLF1** | 1549-F | AAAACTCGAGATGGGTCGACGATGCGCGCTC | 31 |
|  | 1549-R | CGGGCATATGGACCATGCCAAACTCACTCGAAGCT | 35 |
| **ompA** | 2522-F | AAAACTCGAGGATGCGATCGATTCGCGGCTTACTG | 35 |
|  | 2522-R | CACTCATATGCCTGCGGTAATCTCAATTTCGAGAGGAG | 38 |
