# Supplementary material for: Proteomic-based identification of novel EV-derived protein antibodies biomarkers for melioidosis diagnosis
Source: PLoS Negl Trop Dis. 2025 Sep 24;19(9):e0013543. doi: 10.1371/journal.pntd.0013543 (PMC12459824; doi:10.1371/journal.pntd.0013543)
Supplement: S5 Table — (DOCX) [file pntd.0013543.s016.docx]

**S5 Table. The serum samples used in this research**

| **Group** | **Sample** | **Number** |
| --- | --- | --- |
| **experimental group (Positive)** | Melioidosis patients | 43 |
| **control group (Volunteers, Negative)** | Volunteers from non-endemic areas | 47 |
| **Other bacterial infection group (Specificity)** | *K. pneumoniae* infection serum | 5 |
|  | *P. aeruginosa* infection serum | 5 |
|  | *A. baumannii* infection serum | 4 |
|  | *S. maltophilia* infection serum | 3 |
|  | *E. coli* infection serum | 4 |
| **Mass screening** | Convalescent | 5 |
|  | asymptomatic volunteers from endemic areas  (Naturally-Acquired Volunteers Individuals) | 95 |
|  | Laboratory partners, High-Risk Population | 14 |
|  | Farm labourers (High-Risk Population of natural focus; Animal-derived volunteers personnel) | 11 |
| High-Risk Population: Residency in melioidosis-endemic areas (regions with confirmed *Bp*-positive case reports); Occupational exposure (e.g., laboratory partners);  Naturally-Acquired Volunteers: Individuals with environmental exposure to *Bp* through: Occupational contact with contaminated soil/water (e.g., farmers, veterinarians); Residence in endemic regions (Hainan, Guangdong, Guangxi).  Animal-derived volunteers: personnel with direct animal contact in outbreak settings: Farm workers handling *B. pseudomallei*-infected livestock; Veterinarians managing clinical cases Required ≥6 months daily exposure prior to enrollment  Laboratory partners: All research personnel in our team involved in *Bp*-related experiments. | | |
